# Considerations towards the better integration of epidemiology into quantitative risk assessment

**DOI:** 10.1016/j.gloepi.2022.100084

**Published:** 2022-09-09

**Authors:** Sandrine E. Déglin, Igor Burstyn, Connie L. Chen, David J. Miller, Matthew O. Gribble, Ali K. Hamade, Ellen T. Chang, Raghavendhran Avanasi, Denali Boon, Jennifer Reed

**Affiliations:** a Health and Environmental Sciences Institute, Washington, DC, United States of America; b Department of Environmental and Occupational Health, Drexel University, Philadelphia, PA, United States of America; c U.S. Environmental Protection Agency, Washington, DC, United States of America; d Department of Epidemiology, University of Alabama at Birmingham School of Public Health, Birmingham, AL, United States of America; e Oregon Health Authority, Portland, OR, United States of America; f Center for Health Sciences, Exponent, Inc., Menlo Park, CA, United States of America; g Syngenta Crop Protection, LLC., Greensboro, NC, United States of America; h Corteva Agriscience, Indianapolis, IN, United States of America; i Bayer Crop Science, Chesterfield, MO, United States of America

## Abstract

Environmental epidemiology has proven critical to study various associations between environmental exposures and adverse human health effects. However, there is a perception that it often does not sufficiently inform quantitative risk assessment. To help address this concern, in 2017, the Health and Environmental Sciences Institute initiated a project engaging the epidemiology, exposure science, and risk assessment communities with tripartite representation from government agencies, industry, and academia, in a dialogue on the use of environmental epidemiology for quantitative risk assessment and public health decision making. As part of this project, four meetings attended by experts in epidemiology, exposure science, toxicology, statistics, and risk assessment, as well as one additional meeting engaging funding agencies, were organized to explore incentives and barriers to realizing the full potential of epidemiological data in quantitative risk assessment. A set of questions was shared with workshop participants prior to the meetings, and two case studies were used to support the discussion.

Five key ideas emerged from these meetings as areas of desired improvement to ensure that human data can more consistently become an integral part of quantitative risk assessment: 1) reducing confirmation and publication bias, 2) increasing communication with funding agencies to raise awareness of research needs, 3) developing alternative funding channels targeted to support quantitative risk assessment, 4) making data available for reuse and analysis, and 5) developing cross-disciplinary and cross-sectoral interactions, collaborations, and training.

We explored and integrated these themes into a roadmap illustrating the need for a multi-stakeholder effort to ensure that epidemiological data can fully contribute to the quantitative evaluation of human health risks, and to build confidence in a reliable decision-making process that leverages the totality of scientific evidence.

Epidemiological evidence is often a core element informing regulatory risk assessment, thereby necessitating consideration of how best to appraise the quality of the reports of epidemiological studies [1,2]. The London Principles, published in the mid-1990s, could be considered the precursors of a series of frameworks aimed to address this matter [3]. These frameworks include STROBE (STrengthening the Reporting of OBservational studies in Epidemiology), GRADE (Grading of Recommendations, Assessment, Development and Evaluations), HONEES (Harmonization of Neurodevelopmental Environmental *Epidemiology* Studies), and BEES-C (Biomonitoring, Environmental Epidemiology, and Short-Lived Chemicals), which list various criteria characterizing epidemiological study quality and good research practices, and aim to harmonize data reporting [[4], [5], [6], [7]]. Similar frameworks, such as COHERE (Checklist for One Health Epidemiological Reporting of Evidence) and STREGA (STrengthening the REporting of Genetic Association Studies), are designed to guide the reporting of epidemiological data. Although some of these frameworks have been broadly embraced in some areas of research, such as clinical epidemiology, there is an open question about the value of adopting these instruments for the purpose of increasing the useability of human data from environmental epidemiology in quantitative risk assessment [8]. Without being too prescriptive, various guidance and best practices documents have also been published to improve the integrity, value, and transparency of epidemiological research, increase the accountability of researchers, and conserve research resources, thus demonstrating the desire to see environmental epidemiology more fully used to guide evidence-based public health policies [[9], [10], [11], [12]].

Today, toxicology remains the central component for regulation and public health guidance with respect to environmental hazards. However, there is growing concern about the ability of laboratory animals, particularly rodents, to accurately predict human outcomes [13,14]. Rapid progress in various fields of science and technology has generally augmented epidemiological research practices. For example, biomarkers of exposure and effect could enable epidemiology studies to play a greater role in the characterization of exposure-response relationships at environmentally relevant doses and make them a stronger asset in quantitative risk assessment [15].

In 2017, the Health and Environmental Sciences Institute (HESI) launched a technical committee (Environmental Epidemiology for Risk Assessment Committee; “the committee” hereafter) with a mission to understand 1) how epidemiology is used for regulation and public health guidance, 2) how to more fully utilize and leverage environmental epidemiology in quantitative human health risk assessment, and 3) how to promote greater understanding among epidemiologists of the policy process and the data it needs.

Here, we summarize the key information that emerged from some of the committee's activities and articulate our ideas to help improve how human data inform quantitative health risk assessment and decision making.

## 1 Method

In 2018 and 2019, the committee convened experts in risk assessment, epidemiology, exposure science, and statistics from academia, government, non-governmental organizations, and industry in a series of four in-person meetings with four distinct groups of 10–15 people each. Participants were chosen based on their type and level of expertise, track record in their field, and peer recommendation; we did not randomly sample among all potential participants. The number of attendees was kept small to maximize interactions, and each meeting generally lasted about 6 h. The purpose of the meetings was to explore how to realize the potential of environmental epidemiology in regulatory risk assessment and public health decision-making. A fifth meeting was held virtually in 2020 with four representatives of funding agencies. The desired outcome was to investigate how to improve study design, as well as the reporting and sharing of human data, so that they meet the standards of the practice of epidemiology and further contribute to quantitative risk assessment.

Prior to each meeting, participants were asked to answer a set of questions designed by the committee to prime the conversation (Supplemental Material S1). Questions were crafted to establish a baseline regarding how much participants knew about others' fields of expertise (i.e., how much epidemiologists knew about risk assessment and how risk assessors perceived the work of epidemiologists), what their expectations were with respect to colleagues working in other disciplines, and the level of interdisciplinary collaboration. For the fifth meeting, pre-workshop questions with funding agencies revolved around the fundamental aspects of successful proposals, the selection and prioritization of research topics to be funded, and the evaluation and improvement of the impact of funding (Supplemental Material S2). The first part of each workshop was used to discuss and share views and opinions on these topics.

In the second part of each workshop, participants were asked to work on a case study based on three different epidemiological studies of a chemical, the name of which was not disclosed; the studies were selected to include a case-control study, a case-control study nested in a cohort study, and a cohort study (Supplemental Material S3 and S4). Participants were asked to reflect on the study designs and results, and to expand on how much confidence they would place in each study and their individual and combined findings. Participants were also asked to propose potential improvements to the design of each study and reporting of the results. The goal of this exercise was to stimulate a conversation leading to better understanding of which aspects of study design, data analysis, and data reporting were deemed valuable or not.

The third part of each meeting was an open discussion regarding barriers to the better integration of epidemiology into human health risk assessment, how to make progress in that area, what potential incentives could be, and how to develop a path towards the full realization of human data in quantitative risk assessment. Themes and concepts from each discussion were recorded. Direct quotations were recorded from the pre-workshop questions only.

## 2 Lessons learned

The meetings promoted an exchange of ideas that helped bring to light challenges to the application of epidemiology in human health risk assessment. These meetings confirmed the lack of widespread uptake of many recommendations and guidelines published over the past three decades that would assist regulators and decision-makers in better integrating epidemiology into these processes, indicating that a different approach may be needed to stimulate changes in the field if the goal is to better utilize epidemiological findings in quantitative risk assessment.

We developed an impression that a deep, organic philosophical change was necessary, and that such evolution could occur only with a cross-disciplinary engagement of stakeholders. The key lessons that we learned from the focus groups are summarized below, along with a proposed strategy, detailing how to achieve the goal of increasing the use of epidemiology in human health risk assessment. This strategy was based on the systematic analysis and mapping of the necessary steps to realize change. It presents secondary goals to be achieved and intermediate outcomes that must take place (chronologically and in relation to each other) before progress can occur. It also identifies what actionable steps (or activities/interventions) can be taken, and by whom, to realize these outcomes**.** The strategy was developed by us, not participants in the workshops. We do not claim that these are the only necessary and sufficient steps or processes.

**Fig. 1:**
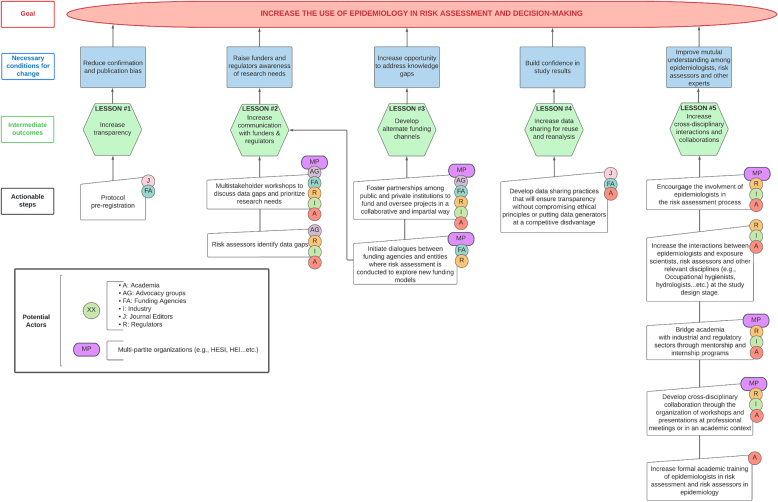
Proposed roadmap towards the more effective use of epidemiology in risk assessment.

Based on our interpretation of the workshop results, we posit that a concerted effort on the part of multiple stakeholders in all activity sectors including academics, funders, regulators, members of industry, representatives of community groups (including trade unions and non-governmental organizations), and journal editors, may be necessary to enact the desired change. The Fig. 1 illustrates the different elements of this change.

### 2.1 Lesson #1: Increased transparency could reduce confirmation and publication biases

People tend to perceive confirmatory tests more favorably than contradictory tests, and therefore are prone to preferentially conduct tests that reinforce rather than negate their beliefs [16]. This human characteristic can blur the distinction between hypothesis generation and hypothesis testing. In other words, despite the commitment of scientists to objectivity and fairness, they may sometimes highlight expected results while downplaying unexpected ones, and modify their initial hypotheses to better fit the observed findings. It has been shown that journal editors, reviewers, and ultimately readers implicitly and perhaps unintentionally reward this practice, hence slowing the advancement of scientific knowledge [17,18]. Similarly, publication bias, which tends to favor publication of positive or (in epidemiology) “statistically significant” findings, may lead to results deemed “negative” to be dismissed as failed or unworthy research, and to never be submitted or published [19]. This pattern is problematic because any research based on a well-defined hypothesis and sound methodology is bound to provide valuable insight.

Workshop participants discussed the influence of confirmation and publication biases in the scientific landscape, as well as ways to reduce them. One proposed solution was to increase transparency through protocol preregistration, which consists of the publication of research hypotheses and methods before any experiments have been conducted [20]. When preregistering a protocol, researchers make public their analysis plans and commit to them without any knowledge of the results. This process could potentially shift the focus to study quality rather than outcomes, and prevent the loss of knowledge due to systematic dismissal of null findings. This practice could be incentivized by funders and journal editors by encouraging (as a matter of editorial policies) the publication of preregistered protocols. The extent to which preregistration achieves desired outcomes would have to be evaluated, as there is currently no guarantee that it would not have adverse unintended consequences, such as stifling creativity and preventing efforts to minimize bias by adapting analytical plans in response to realities of environmental epidemiology, which is never as controlled as a clinical trial or a laboratory experiment [8].

### 2.2 Lesson #2: Increased communication with funders and regulators could raise awareness of research needs

The idea of scientific advancement is often associated with new discoveries and groundbreaking ideas. However, novelty alone is not conducive to the successful pursuit of knowledge. Independent replication of findings is a critical element of the scientific method. In the context of quantitative risk assessment, testing existing hypotheses through new or replicate studies, or even through the reanalysis of datasets, by increasing the robustness of existing observations, can provide risk assessors with the confidence that they need to quantitatively evaluate risks. Testing hypotheses is furthermore recognized as one of the lesser aims of epidemiology, where the recent trends are towards estimation of the magnitude of associations, not on calling them as present or absent based on null-hypothesis testing [21]. However, our dialogue with experts and funders revealed different obstacles to the fostering of research that aims to replicate earlier work and/or obtain less biased effect estimates.

Funding agencies sometimes dedicate (either formally or informally) different pools of money to different types of research on the novelty spectrum, with a small portion of their budget directed towards high-risk/high-reward research. These agencies often reserve larger budgets for research that is less novel and more grounded in, or based on, prior knowledge and research findings [22]. This type of funding could support epidemiology studies that add to an existing pool of data characterizing associations between exposure to environmental contaminants and adverse health effects and provide valuable information for quantitative risk assessments. However, our discussion with funders revealed that they may not always be aware of data gaps or weaknesses in the existing knowledge. Funding agencies engaged in patient advocacy also emphasized that their research funding is driven by stakeholders' demand, and therefore, better informed stakeholders could result in more impactful research.

Generally, increasing funders' awareness of various knowledge gaps and research needs of risk assessors could lead to the creation of new funding opportunities seeking to address them, but traditionally have not been the focus of either investigator-initiated or funder-stimulated research. For example, epidemiology studies evaluating whether earlier reports can be replicated could test the confidence in putative associations between environmental exposures and adverse health effects and support the implementation of policies anchored in robust evidence. This might serve to minimize economic losses and costs and public anxiety and hasten policies and regulations reducing exposures to harmful agents.

This challenge could be overcome by opening or broadening communication channels among funding agencies, regulatory agencies, and other data users. This could be accomplished via the organization of multistakeholder workshops fostering a discussion on existing data gaps, and prioritization of research needs. The goal of such enhanced communication would be to ensure that adequate funding is directed towards research addressing outstanding questions intended to inform policies and regulations.

### 2.3 Lesson #3: Developing alternative funding channels could result in increased opportunities to address existing knowledge gaps

There is a concern that common funding mechanisms of epidemiological research are not designed to explicitly support quantitative risk assessment, but rather favor discovery-oriented research. As such it is not reasonable to expect that existing funding channels will in the future support epidemiological studies that cater to the advancement of quantitative risk assessment, in part due to added cost of such studies [23,24]. Study costs increase with study population size, the accuracy of exposure and confounder evaluation, and quality of outcome assessment. Ultimately, the study design is often determined by the available budget, sometimes at the expense of study quality or size. A careful cost-benefit analysis of study quality versus size has been recommended in the past, and the need to compromise between exposure evaluation and study power was confirmed by epidemiologists who participated in our discussions [25,26].

It was reported during discussions that to increase the chance of getting their proposals funded, researchers may tend to favor maximizing study power calculated under optimistic assumptions of the accuracy of exposure assessment. This may be unconsciously done without the applicants' or funders' awareness or based on a misplaced sense of optimism relating to effect size. However, it could result in not implementing the best attainable, typically costlier, exposure evaluation that might better serve the needs of risk assessment. Because of the high cost of accurate exposure evaluation and other field data collection, this choice is understandable when the applicants seek to increase the chance of their studies being funded. However, it may not always be conducive to maximizing policy impact and may lead to over-confidence in the results (i.e., “adequately powered” may in fact be severely “under-powered” due to exposure misclassification) [[24], [27]].

This challenge could be overcome through the fostering and increased acceptance of “hybrid funding,” such that an investigator receiving money from a public institution or nonprofit organization could apply for supplementary funds from different private or public stakeholders. This funding approach can serve to complement the study by collecting additional data for improved quantitative exposure evaluation that would improve the utility of the study findings for broader risk assessment work. Although historically, some research directly funded by industry has generated controversy, this type of hybrid funding could make private partners into impartial sponsors. Moreover, study outcomes, and potentially the underlying deidentified raw data, would be subjected to review by all funding parties, hence fostering trust that arises from transparency, including explicitly articulated and debated concerns (if any). There are encouraging precedents of work widely accepted as high quality that was supported through such a hybrid mechanism (e.g., the cohort study of European asphalt/bitumen workers, and the whole body of work funded by the Health Effects Institute (HEI)) [28].

HEI can serve as a successful model that could be replicated in different areas of research because it is a highly regarded independent public-private partnership that receives half of its funding from the U.S. Environmental Protection Agency and half from the worldwide automotive industry. This partnership has been successful in funding and overseeing air pollution research for decades. Several participants in our discussions agreed that they would consider receiving public or private funds through a third party or industry consortium with independent oversight.

It would be helpful to initiate a conversation between funding agencies and entities where risk assessment is routinely conducted (e.g., regulatory agencies and industry) to explore how to develop new funding models or increase the use of seldom used ones (e.g., hybrid or pooled funding) that would increase research resources and help epidemiologists conduct thorough studies that might be better able to meet quantitative risk assessment needs. Opening these communication channels would also provide an opportunity for risk assessors to communicate their needs to funding agencies and potentially result in funding opportunities to address existing knowledge gaps. Increased communication with the stakeholders who drive the research in advocacy groups would fulfill the same purpose.

### 2.4 Lesson #4: Increasing data sharing for reuse and reanalysis could build confidence in study results

Over the past decade, public and private funding entities have promoted and implemented open data policies, resulting in the development of many publicly accessible data repositories, especially in the fields of biomedicine and behavioral sciences (e.g., the National Institutes of Health: https://sharing.nih.gov/) [29,30]. For example, the Center for Open Science at the University of Virginia has promulgated a series of Transparency and Openness Promotion (TOP) Guidelines that are meant to encourage this, requiring at the highest level of stringency, that data, code, and ancillary or supporting information be placed in a data repository [31]. Data sharing and transparency are often touted as key to increase replicability and reproducibility, optimize return on research investments, and ultimately promote scientific advancement. However, effective data sharing benefits from context of the information (e.g., why and how they were collected and treated) to allow for appropriate reanalysis or integration to other datasets [32]. Although data sharing is increasingly encouraged and even required by certain funding agencies and journals, our conversation with workshop participants revealed that the practice is not necessarily either common or enforced (where mandated) [33]. It also appears that datasets made publicly accessible remain seldom used, because of subpar data curation and lack of information on data collection [34,35]. While the editors of *Epidemiology* have expressed concerns with the Center of Open Science's TOP Guidelines and provided rationale for these reservations, they do “encourage authors to make their research data and computing code available for replication endeavors”; the editors further state that they “intend to soon ask authors of each new submission to explain whether and how these materials might be made available” [36].

Other reasons why data sharing or release is not universally endorsed might include subpar data curation (as mentioned above), lack of resources, funding, and/or recognition for doing so, could give what are perceived as unfair advantages to others in any future competitive grants or funding applications. In addition, there is concern that the shared data might not be used or interpreted correctly and/or might be re-analyzed to produce contradictory results. Regarding this latter point, although many agree that having data re-analyzed by independent teams enhances trust and confidence, particular concern has been expressed by some that industry and its allies may use these data for nefarious purposes in ways harmful to science. There are many legitimate ways to analyze data but also a great number of illegitimate ways as when (for example) a specific end goal or conclusion is sought. Access to raw data might encourage some with vested interests in the outcomes to “tailor” analysis to these outcomes or at least cause confusion or uncertainty about earlier claimed study results. Some see these efforts reflected in the Secret Science Act or HONEST Act or the now retracted “Strengthening Transparency in Regulatory Science,” proposed by EPA in 2018, which would have required EPA to ensure that the “data and models underlying pivotal regulatory science be publicly available in a manner sufficient for independent validation”. However, one may hope that collaborative (as opposed to “independent”) re-analysis may gain traction in the future, as a guard against unfair and unethical data manipulation [37].

There are important reasons why data sharing is not universally endorsed among environmental epidemiologists, including concerns for data sovereignty at the person and community levels [38]. Similarly, there may be concerns that – despite being de-identified – it may be possible for others to “re-identify” released data, given enough effort, time, ability, and interest. The Belmont Report's bioethical principles of beneficence, justice, and respect for persons must be reflected in all epidemiological research [39]. Data sharing should, of course, be subject to the consent statement signed by the research participants and broader use or reanalysis plans should be considered a priori and incorporated in consent statements. Failing to do so has legal implications, and can lead to breaches of community trust in research. Ideally, and especially when research is associated with specific communities, researchers would engage these communities in data analysis and presentation so that the outcome considers nuances that might not be evident solely from researchers' perspective. It would also give community ownership and confidence in the research and outcomes.

Some meeting participants were in favor of open data policies, suggesting that funding agencies and journals could play a role in ensuring that open data practices are more widely adopted. Funders can require that de-identified study data are made public, but adequately funding and enforcing that policy could be challenging. Journals can also require a statement addressing data access policies, which may include direct data sharing, as a condition of publication; again, enforcement is problematic. There often is reluctance to complete openness on the part of researchers who have invested a substantial amount of time and resources into generating data and curating results in addition to privacy concerns, which often prevent data sharing. To overcome this issue, certain journals have adopted the Joint Data Archiving Policy (JDAP), which allows authors to embargo their data for up to a year, or more if sufficiently justified [40]. Although a longer (or in certain cases to protect privacy, permanent) embargo might be appropriate in the case of environmental epidemiology, this model could potentially help release data to the public domain, if there is material support for the costly effort of curating data, and if protection of personal health information can be ensured. Just as with the idea of registration of study protocols, the impact of policies of “open data” within the context of environmental epidemiology appears to deserve a careful study as one would with any intervention, focusing on benefits and risks of specific implementations, instead of embracing the abstract idea wholesale. The current experience with the proposition of “open data” in epidemiology has not been a resounding success and has drawn some well-articulated concerns from leading epidemiologists and journal editors [8].

Interestingly, giving study authors the opportunity to signal their adoption of open practices in science appears to be an incentive not only to share data but also to ensure that these data are well curated and useable. This was demonstrated by the journal *Psychological Science*, which after adopting an “Open Data” badge system in 2014, saw an increase in reporting of open data from a 3% baseline to 39% in the first half of 2015. The badges were awarded based on criteria defined by the Center for Open Science (www.cos.io) which promotes open research and offers various platforms to share data, software, and other materials, as well as preregister research and share publications and pre-prints [41]. Today, over 75 journals, most of them in the behavioral sciences, offer open science badges. Authors who apply for badges upon article acceptance must provide evidence that all data supporting their study are shared in an open-access repository. The awarded badges are then displayed at the top of the publication, and a URL to the data is provided. Similar practices could be tested in the field of environmental epidemiology, but they will require the buy-in of journals and funding agencies in incentivizing and empowering researchers towards open data. It is unclear whether epidemiologists will be motivated by such badges, and this remains a topic worthy of an in-depth examination.

Alternatively, a “half a loaf” approach may be considered by which the raw data are not directly released, but sufficient information is released to increase confidence in the results. For example, the article “A Pragmatic Approach for Reproducible Research with Sensitive Data” and the accompanying commentary “Reproducing Epidemiologic Research and Ensuring Transparency” appearing in the American Journal of Epidemiology introduces the concept of “quasi-reproducibility” [42,43]. Research is considered to be quasi-reproducible if “analysis code, simulated data sets, and reports applying analysis code to the simulated data are posted on a publicly available website and the code can be run by independent researchers to obtain identical results [*to the simulated data sets*]”. The authors acknowledge that “this is not a perfect solution, … [*but it is*] a suitable comprise between reproducibility and data protection.” Advantages of this approach include the ability to make data transparent for critical evaluation and dissemination, enhancing confidence in research, reducing ambiguity regarding statistical and other analyses and analytical approaches, and allowing others to benefit from already developed code. From a policy perspective, utility of the research for decision-making would be enhanced if additional information was made available. Such additional information for epidemiology studies might include, for example,

• Actual output from the code applied to the true data set;
• How and why the covariates or confounders were selected (e.g., Directed Acyclic Graphs, or DAGs; model fitting; professional opinion or expertise, previous research) and how the number of predictors and parameters relates to the number of events;
• Presentation of not just adjusted odds ratios, but crude odds ratios and perhaps intermediate or minimally adjusted ratios too;
• Robustness of results to alternative assumptions, including sensitivity analysis and/or quantitative bias analysis and/or adjustments for biases (e.g., via Bayesian techniques).

While the above may not satisfy those that feel that a full release of raw data is a prerequisite for adequate understanding of the data and full transparency, it may be enough to considerably increase the confidence in the results and conclusions by giving the reader or reviewer better access to the methods, techniques, and thought processes of the investigators. It also has the advantage of allowing the reader or reviewer to ask better and more focused questions of the researchers and to facilitate such conversations.

### 2.5 Lesson #5: A lack of sufficient cross-disciplinary interactions and collaboration is a barrier to better integration of epidemiology in risk assessment and decision-making

During our meetings, discussions made apparent that the lack of cross-disciplinary collaboration was a barrier to the integration of epidemiology in risk assessment and decision-making. Assessing the risks associated with environmental chemicals requires a broad range of expertise, determined by the nature of the problem, and, in some cases, not limited to the already wide range of the core disciplines that include exposure sciences, toxicology, statistics, epidemiology, and risk assessment. However, we observed that scientists working in even the seemingly core fields are often siloed. Communication and collaboration are conducive to transfer of knowledge and learning and are likely to increase robustness and policy-relevance of research. Increased interactions with risk assessors (including involvement, perhaps even as observers in regulatory risk assessment) could help epidemiologists build on their understanding of risk assessment to better tailor their research and make it more impactful in the realm of quantitative risk assessment. In addition, open dialogue with exposure scientists could provide valuable information at the study design stage [12,44]. Risk assessors could also benefit from direct interactions with epidemiologists to better understand how the research was conducted, and consequently better apply study results to their evaluation and decision making. Risk assessors may learn to trust and better appreciate the caveats of epidemiology by actively collaborating on epidemiological studies (where this does not create a conflict of interest). Integration of other disciplines into discussion motivated by specifics of a given problem appears to likewise offer considerable benefits (e.g., occupational hygiene when exposures are occupational, hydrology when exposures are water-borne).

It would also be helpful to bridge the academic sector with other sectors, such as industry and government, by fostering mentorship and internship programs that could give epidemiology students the opportunity to discover how their influence can have a positive impact on these professional environments.

Cross-disciplinary collaboration may produce research that is more seamlessly applicable to informing environmental health policies. This could be achieved via the organization of interactive workshops and presentations, either at professional meetings or in the academic context, where up-and-coming and seasoned epidemiologists alike could better understand regulatory risk assessment. Increased networking among the different disciplines would also help break existing silos.

Such cross-disciplinary collaborations may be best fostered through cross-disciplinary training. Our discussions with experts also revealed that epidemiologists rarely receive formal training in regulatory risk assessment (or risk assessment in general) and consequently, are not necessarily aware of the needs of regulatory agencies in design of their studies. Although risk assessors seem to have some background in epidemiology, their knowledge can remain insufficient to effectively interpret epidemiological studies and better integrate those findings into decision-making processes. Participants in our discussions generally agreed that more cross-disciplinary training would be beneficial to students by better preparing them to recognize and address knowledge gaps and ultimately ensure that their research will be used in practice. One example of such a need was emphasized by the National Academies of Sciences, Engineering and Medicine in their 2019 report on reproducibility and replicability in science, which suggested that reproducibility could be improved if industry and other entities, such as national laboratories, offered training in statistical analysis and inference [45]. The manner by which cross-disciplinary training can be fostered is beyond scope of the meetings, but we speculate that internships with regulatory agencies and other users of epidemiological data during graduate training may be effective.

## 3 Conclusion

The discussions hosted by the committee identified five key needs that may increase the impact that epidemiological studies have on quantitative risk assessment, and ultimately improve the relevance and utility of the risk assessment. Increased transparency, open communication with funders, improved data sharing, creation of new funding channels, and more inter-disciplinary collaboration and cross-training could help solve the longstanding problem that epidemiology is under-used in regulatory risk assessments, and in turn, policy making.

Some of the proposed measures would benefit from increased collaboration among scientists of all sectors and disciplines related to epidemiology and risk assessment. Organization such as HESI are well positioned to bring together professionals from various fields and sectors who usually seldom interact, and provide a neutral platform for them to engage on common issues.

For example, HESI's Environmental Epidemiology for Risk Assessment Committee could help facilitate the conversations between funding agencies and regulators and foster the development of short courses on how to conduct epidemiology studies that have more weight on and utility for risk assessment and the decision-making process.

In the pursuit of an educational purpose, the committee is currently hosting a series of free monthly webinars illustrating the use of epidemiology in risk assessment (https://hesiglobal.org/environmental-epidemiology-for-risk-assessment/). Additionally, the committee recently developed a web platform that will serve as a resource of information relevant to epidemiology and risk assessment (www.epifora.org). This site also hosts a searchable database of professionals and students in the field and serves as a hub for this community of practice. Users can search for collaboration opportunities, internships, mentorship opportunities, and can establish a network beyond the scientific silo in which they commonly operate. Our discussions with stakeholders indicate that to have a lasting positive impact on evidence-based environmental health policies, next steps in the application of epidemiology to quantitative risk assessment will be best done as a collaborative endeavor committed to change over long timescales.

## Appendix A Supplementary data

Pre-Workshop Questions Image 1

Pre-Meeting Questions (Funding Agencies) Image 2

Case Study Presentation Image 3

Case Study Presentation (Funding Agencies) Image 4
