# Supplementary material for: Considerations towards the better integration of epidemiology into quantitative risk assessment
Source: Glob Epidemiol. 2022 Sep 9;4:100084. doi: 10.1016/j.gloepi.2022.100084 (PMC10445996; doi:10.1016/j.gloepi.2022.100084)
Supplement: Supplementary file 1 — Pre-Workshop Questions [file mmc1.pdf]

HESI

Pre-workshop Questions

Depending on whether you are an epidemiologist or a risk assessor, could you please answer the questions below. These will help frame the discussion the day of the meeting.

For epidemiologists

1. **When you are designing a study, to what degree are you thinking about how the results will be used by a risk assessor or in regulatory decision making?**

2. **What would help increase your knowledge of what risk assessors or regulatory decision makers need or would find helpful in using or interpreting results from epidemiological studies?**

3. **What do you think are the strongest factors and considerations from epidemiology studies that would be most important or relevant for public health decision making? What are the weakest aspects that can limit the consideration of epidemiological evidence? Which if any of these aspects might you consider to be critical for the epidemiological picture and that you might consider in interpreting epidemiological evidence?**

For risk assessors, decision makers, users of epidemiology

1. **To what extent do you or are you able to incorporate epidemiology studies into risk assessment or decision making? If so, what role does it play, and in what ways is it communicated or interpreted? Are there criteria/guidelines or thought processes that you use? If so, what are they?**

2. **In what ways do you assess the validity and utility of epidemiology studies? Are there criteria/guidelines or thought processes that you use? If so, what are they?**
