# Supplementary material for: Considerations towards the better integration of epidemiology into quantitative risk assessment
Source: Glob Epidemiol. 2022 Sep 9;4:100084. doi: 10.1016/j.gloepi.2022.100084 (PMC10445996; doi:10.1016/j.gloepi.2022.100084)
Supplement: Supplementary file 2 — Pre-Meeting Questions (Funding Agencies) [file mmc2.pdf]

HESI

Pre-Meeting Questions

This set of questions will be shared with participants, and responses will be collected prior to the meeting. Participants will be given between 1 and 2 weeks to answer these questions. Answers will be briefly summarized during the meeting, followed by a discussion. Note that all answers will remain anonymous.

Questions:

- **Essential Information**
  - What are the key characteristics of successful funding proposals (e.g., novelty, potential for application, data gap filling…etc.)?
    - Are there specific major “pluses” you like to see in funding applications and – conversely – major negatives that may limit consideration of an otherwise reasonable proposal?
  - How are proposals seeking qualitative results (e.g. evidence for or against existence of risk from a given exposure) weighted in comparison to those with more quantitative objectives (e.g. how much risk is there from a given exposure?).
  - How important is it in the funding decision, that a proposal provide adequate information to ensure study reproducibility?

- **Essential Practices**
  - To what extent, if any, are data sharing and transparency by the grantees considered in making funding decisions (Supposing they strictly respect privacy protection practices)?
  - Do you require grantees to include a data dissemination plan, as opposed to result dissemination plan, in their proposal?
  - How is study replication (i.e., addressing an issue that has already been studied) weighted compared to more novel research?

- **Filling Information Gaps**
  - How are the research topics to be funded decided?
  - How are existing needs and gaps in public policy and human health risk assessment factored into your funding allocation?

- **Impact of Funding**
  - How do you evaluate the impact of the research you fund?
  - How do you think research agencies can increase the impact of research they fund on human health risk assessment?
