# Supplementary material for: Considerations towards the better integration of epidemiology into quantitative risk assessment
Source: Glob Epidemiol. 2022 Sep 9;4:100084. doi: 10.1016/j.gloepi.2022.100084 (PMC10445996; doi:10.1016/j.gloepi.2022.100084)
Supplement: Supplementary file 3 — Case Study Presentation [file mmc3.pdf]

Application of Environmental Epidemiology for Risk Assessment and Decision Making

Friday December 6, 2019

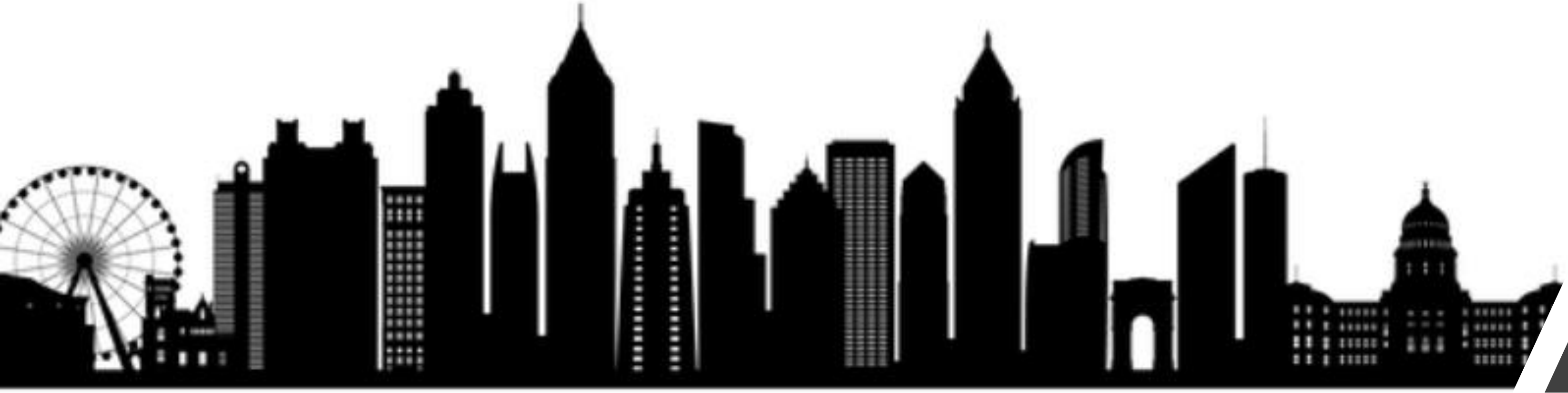

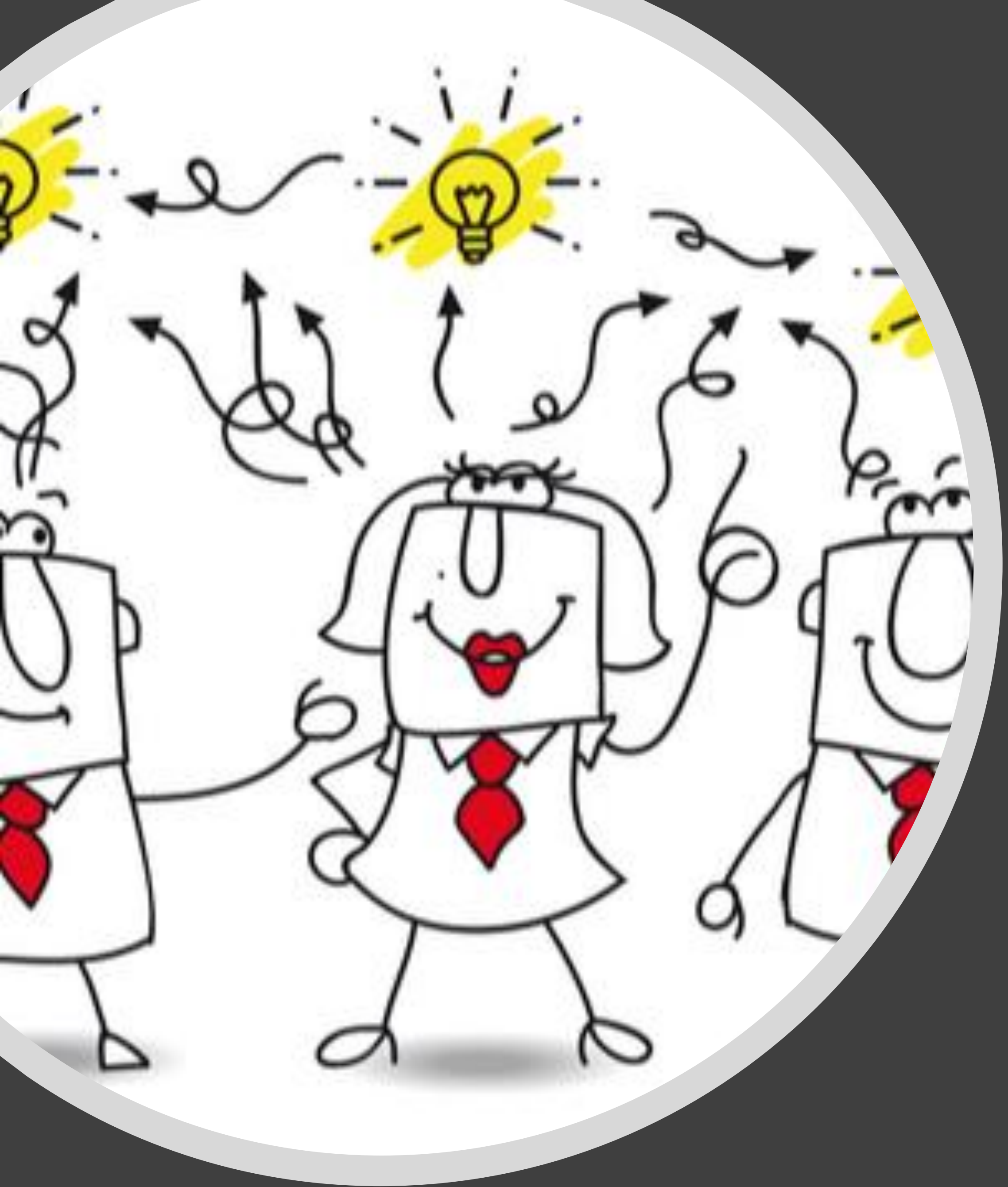

Case-study

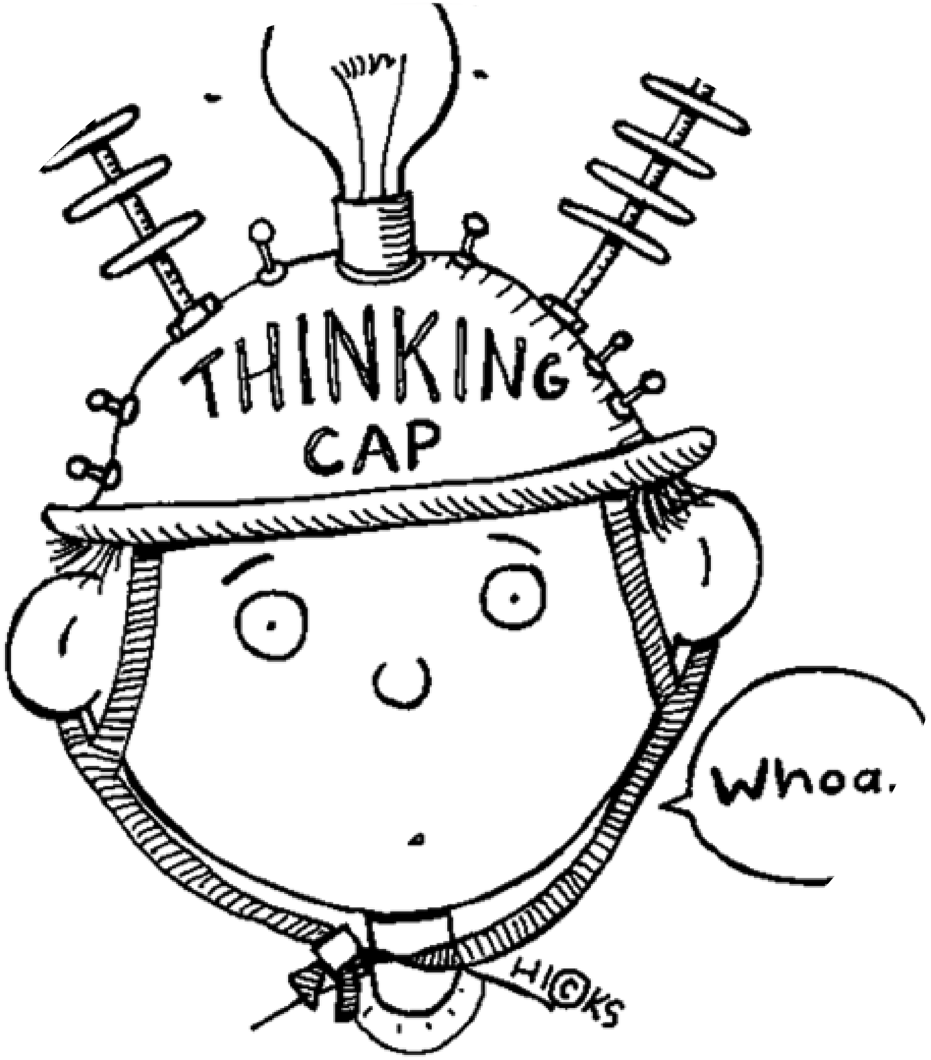

As we proceed through the case studies …

- What is evidence for/against causality?
- What would you do? What would you recommend given the information in front of you?
- What additional information would you need to be confident in your recommendation/course of action?

This Photo by Unknown Author is licensed under CC BY-NC-SA

Case Study: “HESI-X”

HESI-X

Does not occur naturally in the environment

Occupational use only (small amounts detected in food)

Primary exposure route = dermal; secondary = inhalation, ingestion

Rapidly broken down and eliminated via urine. Little bioaccumulation (eliminated ~ 12 days)

No evidence of carcinogenicity in animals (2-year oral cancer bioassay in rodents).

LEUKEMIA

There are three observational epidemiological investigations studying the possible relation between HESI-X exposure and leukemia:

- Case Control
- Ecologic
- Cohort

LEUKEMIA

C-C

**Population/ Case Ascertainment**	**Tumor registries/hospital records**
**Exposure Assessment, Response Rate**	**Interview-based questionnaire** (trained interviewers)<br>• Cases = 578 (340 living); **86%** response rate; **41%** completed by proxy<br>• Controls = 1245; **78%** response rate; **34%** completed by proxy
**OR (95% CI)**	1.2 (95% CI: 0.60 – 2.1) ever vs. never use; **n=17**<br>• **Controlled for relevant confounding factors; frequency-matched**

- Any concerns/comments regarding study design or outcome?
- What is evidence for/against causality?
- What would you do? Recommend?
- What additional information would you need to be confident in your recommendation/course of action?

LEUKEMIA

	C-C	Ecologic
**Population/ Case Ascertainment**	Tumor registries/Hospital records	Tumor registry (linked with occupational cohort)
**Exposure Assessment/ Response Rate**	Interview-based questionnaire (trained interviewers)<br>• Cases = 578 (340 living); 86% response rate; 41% completed by proxy<br>• Controls = 1245; 78% response rate; 34% completed by proxy	**Group-level characterization** of exposure estimated by linking union/job history records<br>• **Number of exposed cases on which OR is not provided (n=51 cases for total leukemia)**
**OR (95% CI)**	1.2 (95% CI: 0.60 – 2.1) ever vs. never use; n=17<br>• Controlled for relevant confounding factors; frequency-matched	1.32 (95% CI: 0.65 – 2.65) high vs. low for all leukemias.<br>• Adjusted for age, sex, length of time in union, date of union membership

- Any concerns/comments regarding study design or outcome?
- What is evidence for/against causality?
- What would you do? Recommend?
- What additional information would you need to be confident in your recommendation/course of action?

LEUKEMIA	C-C	Ecologic	Cohort
**Population/ Case Ascertainment**	Tumor registries/Hospital records	Tumor registry (linked with occupational cohort)	Tumor registry (linked with occupational cohort study)
**Exposure Assessment/ Response Rate**	Interview-based questionnaire (trained interviewers)<br>• Cases = 578 (340 living); 86% response rate; 41% completed by proxy<br>• Controls = 1245; 78% response rate; 34% completed by proxy	Group-level characterization of exposure estimated by linking union/job history records<br>• Number of exposed cases on which OR is not provided (n=51 cases for total leukemia)	**Self-administered questionnaire**
**OR (95% CI)**	1.2 (95% CI: 0.60 – 2.1) ever vs. never use; n=17<br>• Controlled for relevant confounding factors; frequency-	1.32 (95% CI: 0.65 – 2.65) high vs. low<br>• Adjusted for age, sex, length of time in union, date of union membership	No exposure: 1.0 (ref); n=21<br>Exposure T1: 0.99 (95% CI: 0.23, 4.24);**n=2**<br>Exposure T2: **2.46 (95% CI: 0.91, 6.66); n=5**<br>Exposure T3: **2.88 (95% CI 0.92, 9.03); n=4**<br>**p for trend = 0.053**

- Any concerns/comments regarding study design or outcome?
- What is evidence for/against causality?
- What would you do? Recommend?
- What additional information would you need to be confident in your recommendation/course of action?

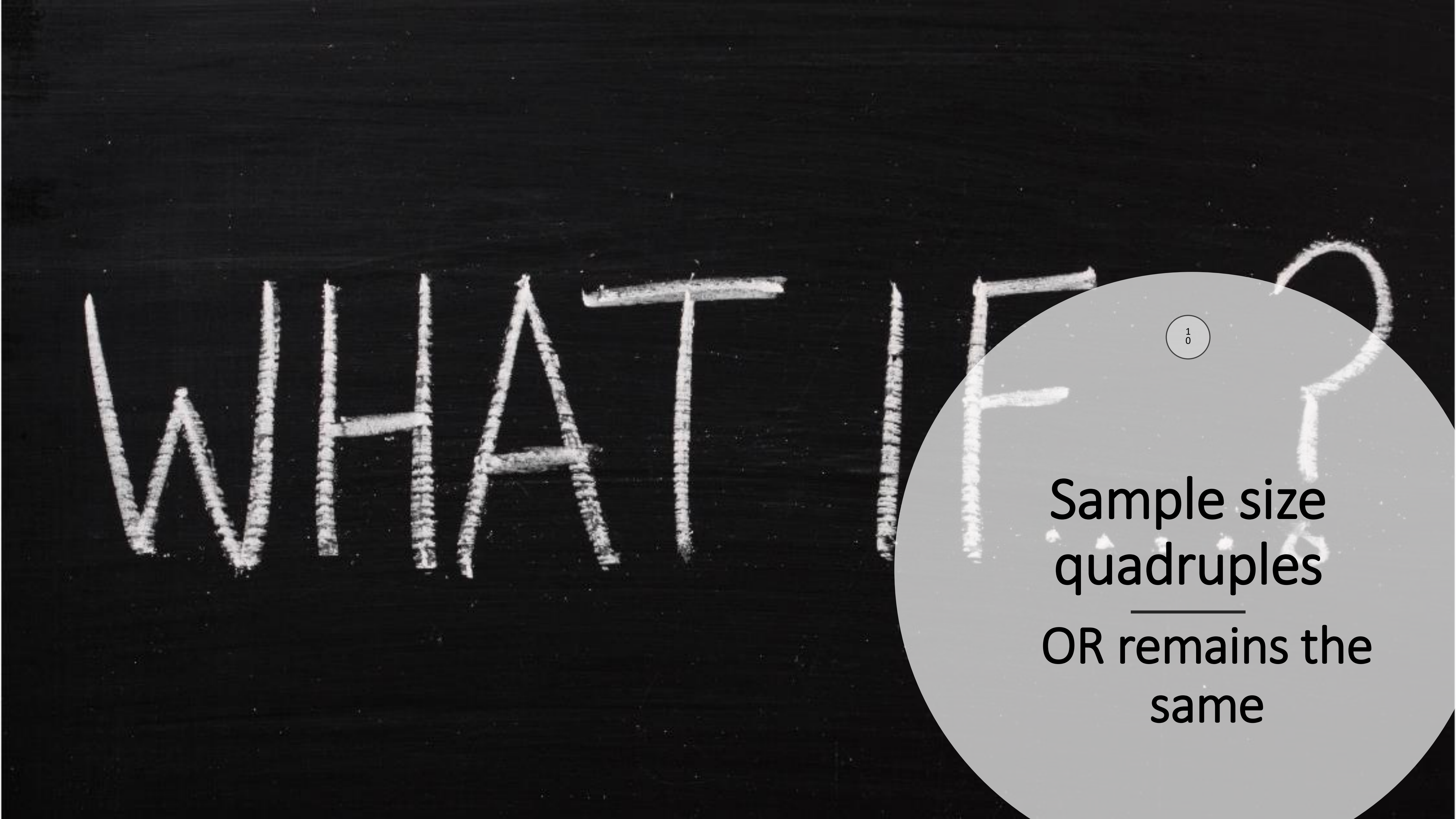

Sample size quadruples

OR remains the same

LEUKEMIA	C-C	Ecologic	Cohort
**Population/ Case Ascertainment**	Tumor registries/Hospital records	Tumor registry (linked with occupational cohort)	Tumor registry (linked with occupational cohort study)
**Exposure Assessment/ Response Rate**	Interview-based questionnaire (trained interviewers)<br>• Cases = 578 (340 living); 86% response rate; 41% completed by proxy<br>• Controls = 1245; 78% response rate; 34% completed by proxy	Group-level characterization of exposure estimated by linking union/job history records<br>• Number of exposed cases on which OR is not provided (n=51 cases for total leukemia)	**Self-administered questionnaire**
**OR (95% CI)**	1.2 (95% CI: 0.60 – 2.1) ever vs. never use; n=17<br>• Controlled for relevant confounding factors; frequency-	1.32 (95% CI: 0.65 – 2.65) high vs. low<br>• Adjusted for age, sex, length of time in union, date of union membership	No exposure: 1.0 (ref); n=21<br>Exposure T1: **0.99 (95% CI: 0.48, 2.05);n=8**<br>Exposure T2: **2.46 (95% CI: 1.50, 4.05); n=20**<br>Exposure T3: **2.88 (95% CI: 1.63, 5.10); n=16**<br>**p for trend = most likely <0.05**

- Any concerns/comments regarding study design or outcome?
- What is evidence for/against causality?
- What would you do? Recommend?
- What additional information would you need to be confident in your recommendation/course of action?

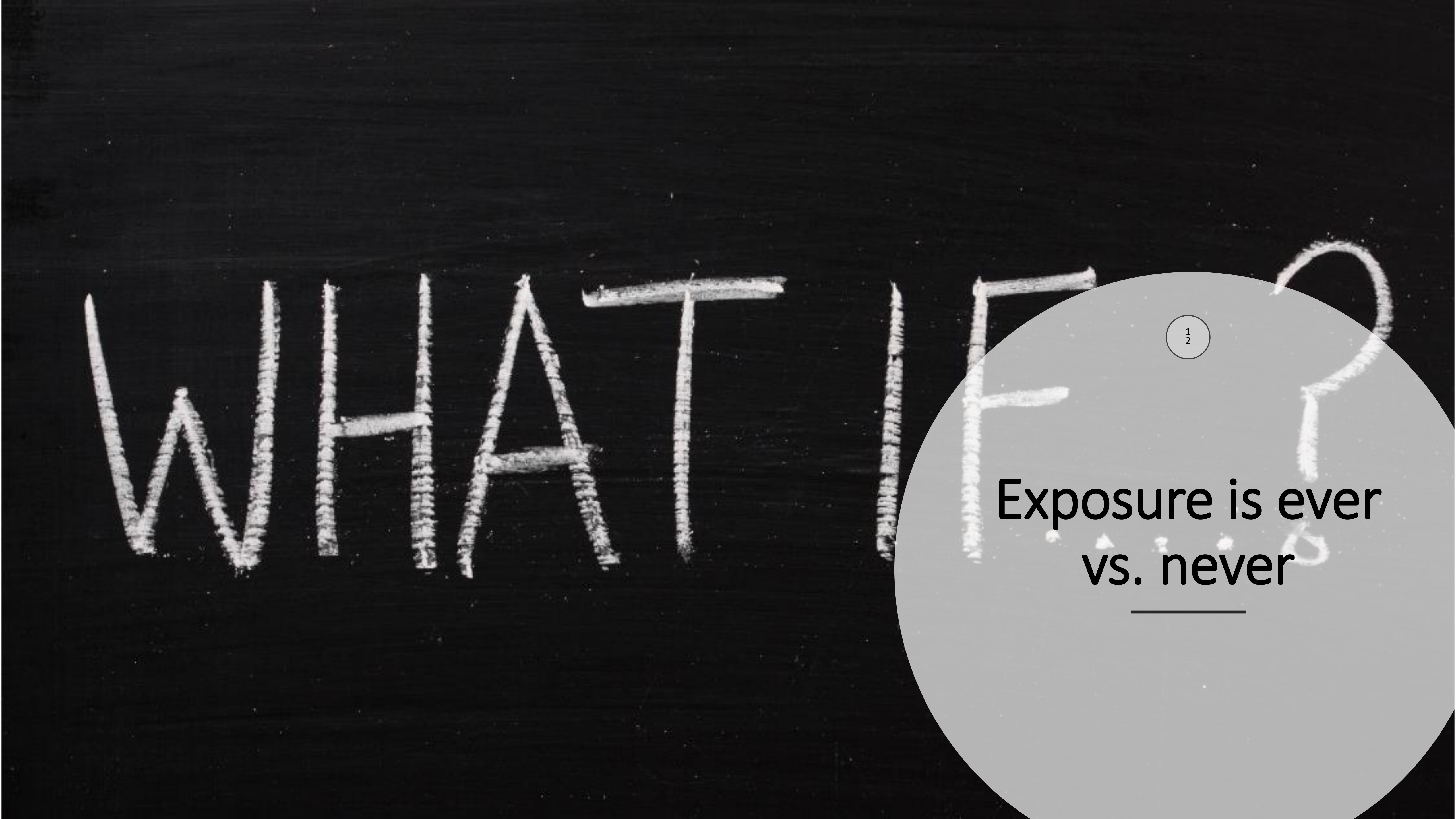

Exposure is ever vs. never

LEUKEMIA	C-C	Ecologic	Cohort
**Population/ Case Ascertainment**	Tumor registries/Hospital records	Tumor registry (linked with occupational cohort)	Tumor registry (linked with occupational cohort study)
**Exposure Assessment/ Response Rate**	Interview-based questionnaire (trained interviewers)<br>• Cases = 578 (340 living); 86% response rate; 41% completed by proxy<br>• Controls = 1245; 78% response rate; 34% completed by proxy	Group-level characterization of exposure estimated by linking union/job history records<br>• Number of exposed cases on which OR is not provided (n=51 cases for total leukemia)	**Self-administered questionnaire**
**OR (95% CI)**	1.2 (95% CI: 0.60 – 2.1) ever vs. never use; n=17<br>• Controlled for relevant confounding factors; frequency-	1.32 (95% CI: 0.65 – 2.65) high vs. low<br>• Adjusted for age, sex, length of time in union, date of union membership	**No exposure: 1.0 (ref); n=21**<br>**Ever : 2.15 (95% CI: 1.10, 4.18); n=11**

- Any concerns/comments regarding study design or outcome?
- What is evidence for/against causality?
- What would you do? Recommend?
- What additional information would you need to be confident in your recommendation/course of action?

LEUKEMIA

Design	Population	Exposure	Outcome	Comparison	N	OR (95%CI)	p-value
**Case control**	Men	Questionnaire	Cancer registry	Reference (No use)	39	1	
				Ever Use	17	1.2 (0.6-2.1)	
**Ecologic**	Workers	Group-level Job records	Cancer registry	Reference (Low exp.)		1	
				High Exposure		1.32 (0.65-2.65)	
**Cohort**	Workers	Questionnaire	Cancer Registry	Reference (No exp.)	21	1	p-trend: 0.053
				Exposure Tertile 1	2	0.99 (0.23-4.24)	
				Exposure Tertile 2	5	2.46 (0.91-6.66)	
				Exposure Tertile 3	4	2.88 (0.92-9.03)	

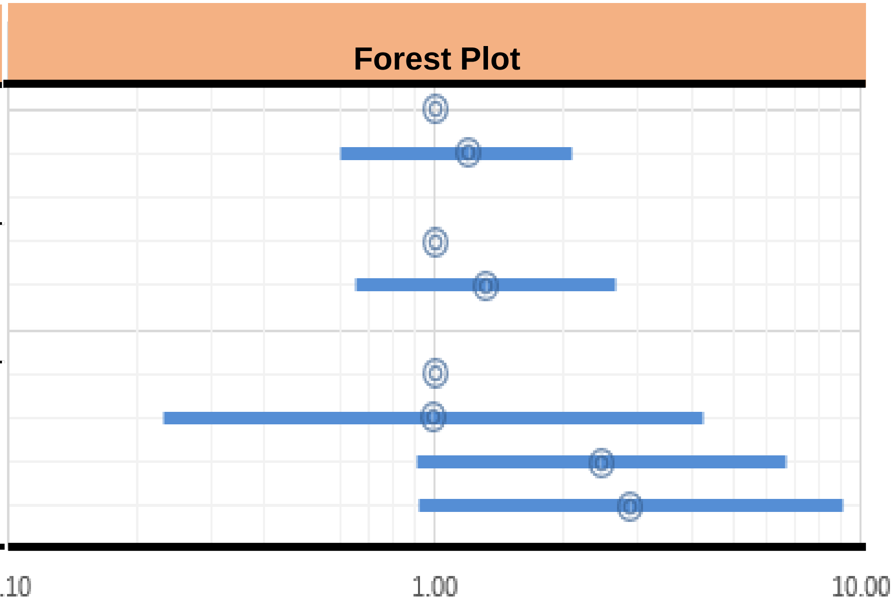

What would you do with this information?

- **Wait for new epi studies?**
- **Additional cohort updates?**
- **More animal experiments?**
- **Investigate MOA/AOP?**
- **Systematic review/meta-analysis?**
- **Regulatory Action?**
  - Cost/benefit?
    - Attributable fraction?
  - Risk management/mitigation
  - Wait/Not wait
    - If not now, when? And wait for what?
- **Something Else?**

Cohort

If sample size quadruples:

OR	LCI	UCI	SE	n cases	sample size increases N times	New SE	New LCI	New UCI	New n cases
0.99	0.23	4.24	0.743428	2	4	0.3717142	0.48	2.05	8
2.46	0.91	6.66	0.507763	5	4	0.2538814	1.50	4.05	20
2.88	0.92	9.03	0.582636	4	4	0.2913181	1.63	5.10	16
