# Supplementary material for: Considerations towards the better integration of epidemiology into quantitative risk assessment
Source: Glob Epidemiol. 2022 Sep 9;4:100084. doi: 10.1016/j.gloepi.2022.100084 (PMC10445996; doi:10.1016/j.gloepi.2022.100084)
Supplement: Supplementary file 4 — Case Study Presentation (Funding Agencies) [file mmc4.pdf]

Application of Environmental Epidemiology for Risk Assessment and Decision Making

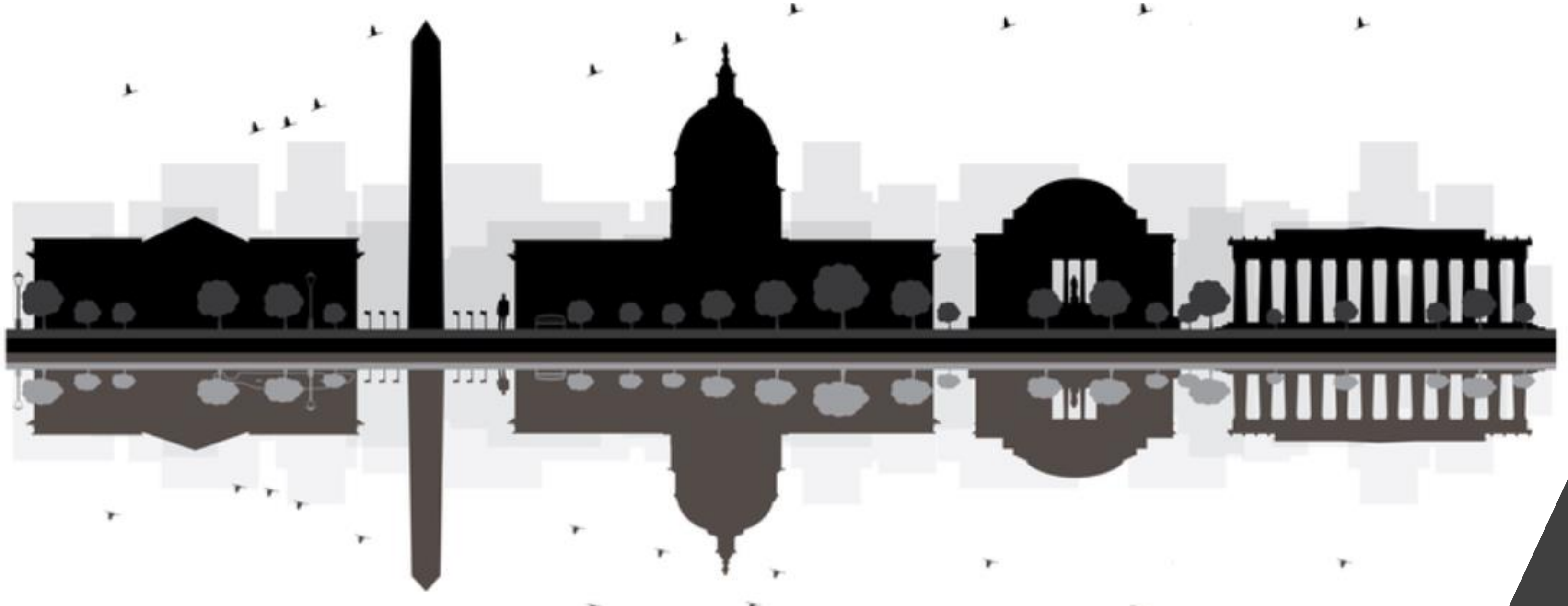

November 23, 2020

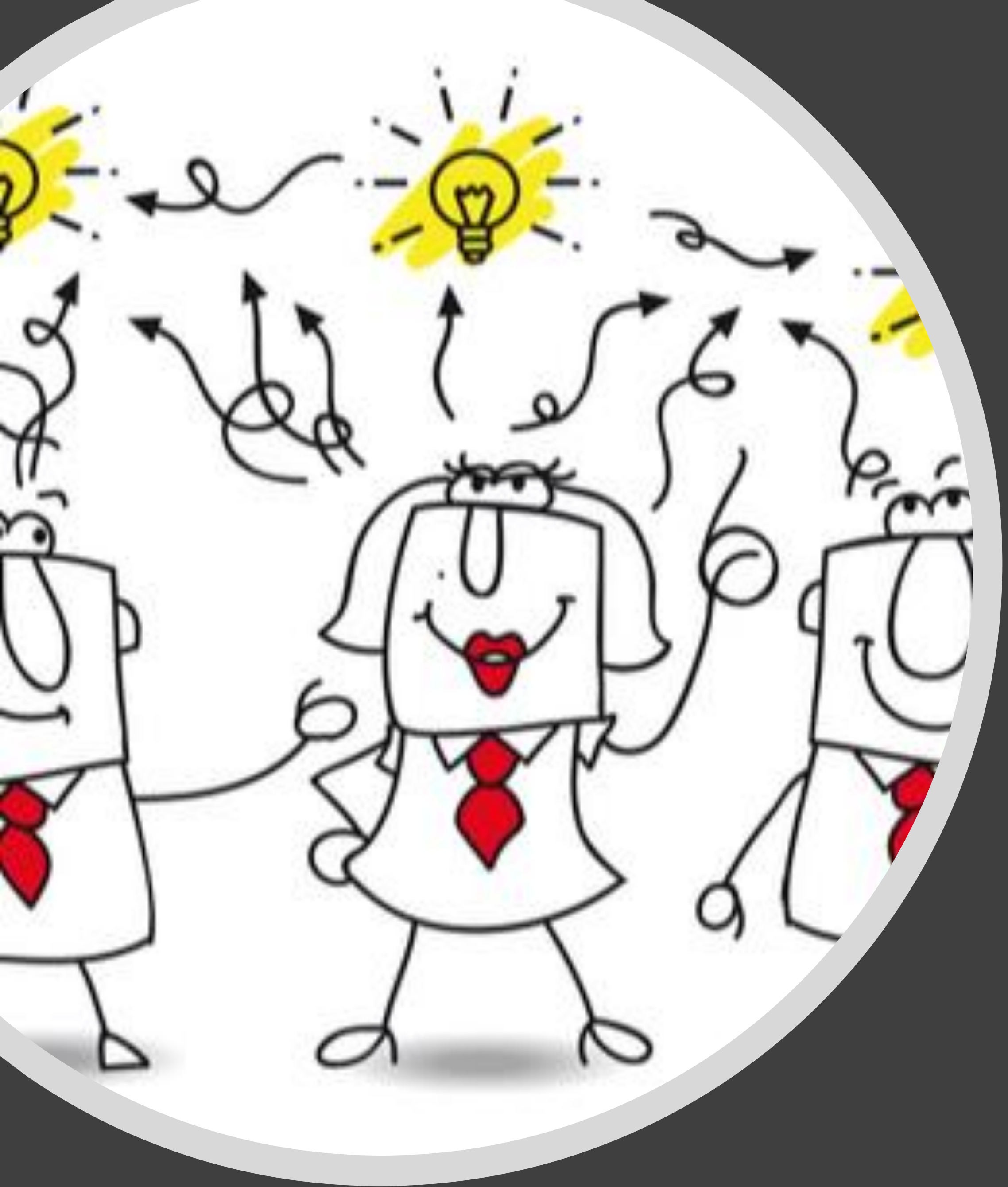

Case-study

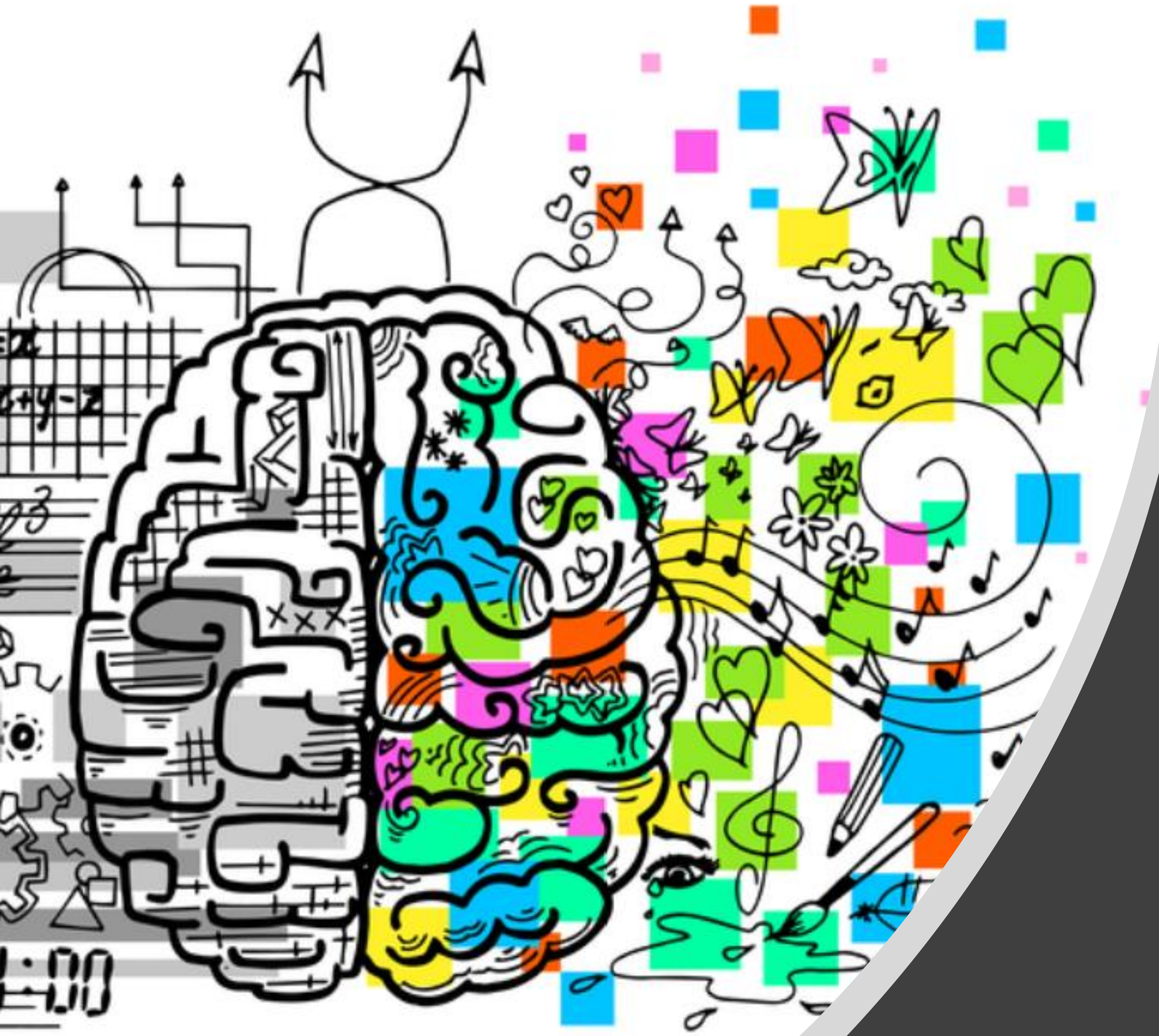

“The best ideas emerge when very different perspectives meet.”
-- Frans Johansson

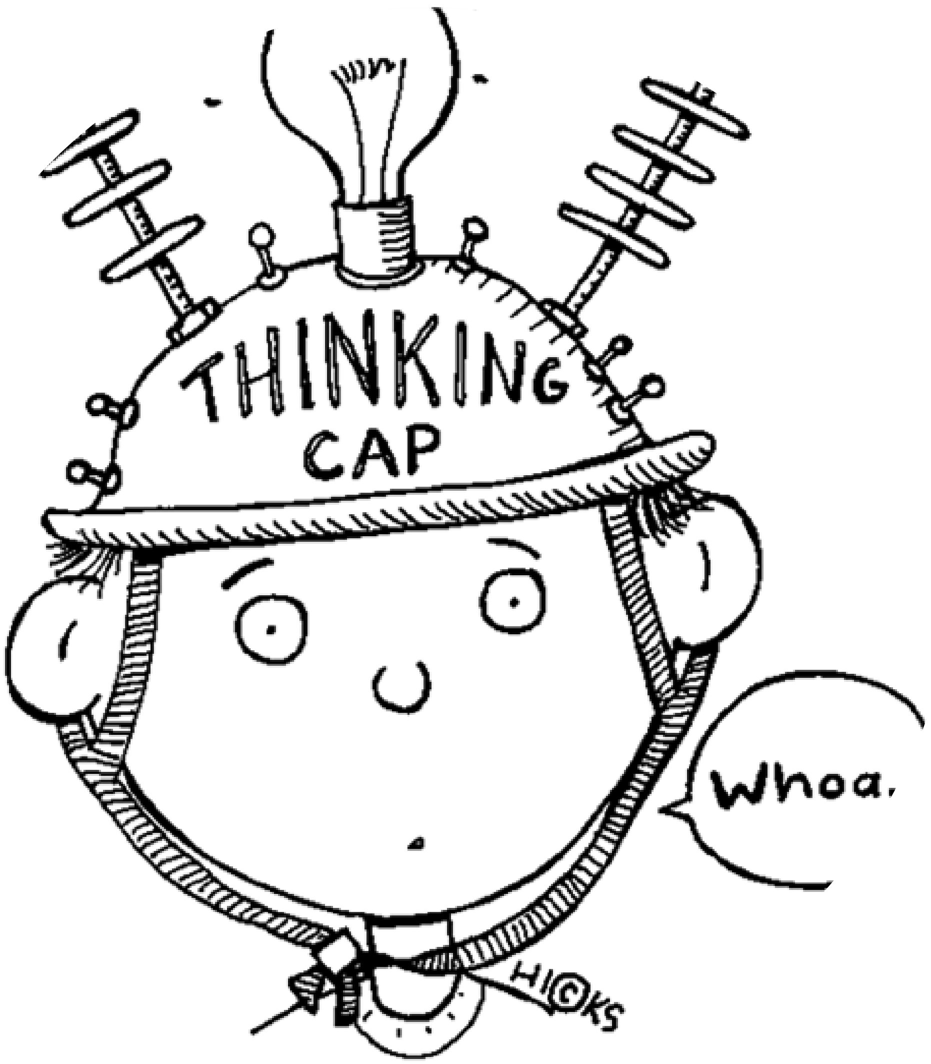

As we proceed through the case study …

- What are important criteria to determine the quality of a proposal?
- What would you decide or recommend given the information in front of you?
- What additional information would you need to be confident in your recommendation/ course of action?
- What about the proposed study designs caught your eye?
- Did you notice anything that is usually not part of research proposals?

This Photo by Unknown Author is licensed under CC BY-NC-SA

Thought Questions

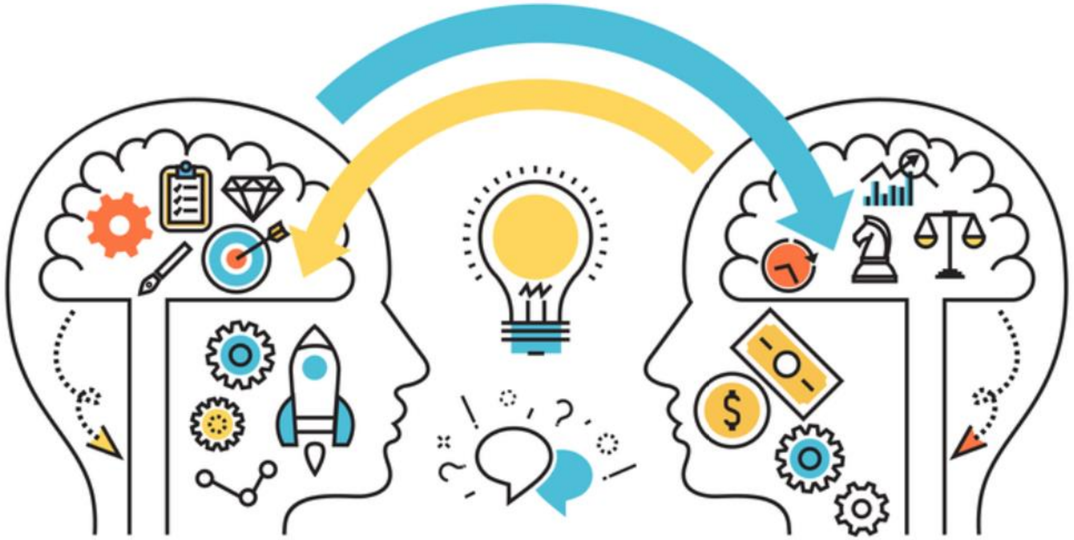

- How do you judge if the **study design is appropriate** and the **sample size adequate** for the research question of interest?
- How do you judge if the **study is adequate to quantitatively assess exposure** with **validity and reliability**?
- How do you judge if the **statistical approach**
  - Is appropriate and adequately **addresses the research question**?
  - Is appropriate and sufficient in addressing **potential bias**?
- Is risk better reported as a **relative risk** (ratio) or as an **additional number of cases**?
- How much does **data transparency and data dissemination** matter in the decision to fund a study?
- How much do **privacy issues** complicate data sharing?

Notice of Funding Opportunity

Originator: Safety-First Fund

Safety First Fund

Occupational Health Protection Initiative

Purpose of the Initiative

Improve occupational health through

- **Awareness of potential risks** to workers
- The **development of evidence-based strategies and policies** that will mitigate these risks, and measurably help prevent disease and injuries in the workplace.

Mission of the Initiative

Minimize workplace illness and injuries by

- **Assessing occupational environments**, to evaluate disease severity and prevalence, exposure to potentially hazardous agents, fatalities, as well as workers and occupations at greatest risk
- **Developing research and prevention strategies**
- **Communicating findings** to stakeholders, and
- **Evaluating the results** of preventative measures

Notice of Funding Opportunity

Expected Impact of Funding

To positively impact occupational safety and health via…

- The **identification and generation of relevant data** that could be used to characterize risk in the workplace, as well as the burden of injuries, illnesses, exposures and fatalities.
- The **prioritization** of prevention, surveillance, intervention, and outreach efforts to address potential occupational risks.
- The **development a network of partners and stakeholders** to assist in these efforts.
- The **development and dissemination of public health recommendations**, interventions and policies that will improve workers safety and health.
- **Effective outreach strategies** to ensure recommendations are understood and appropriately implemented.

Proposed Research on CHEM-X

Three Proposals to Study CHEM-X

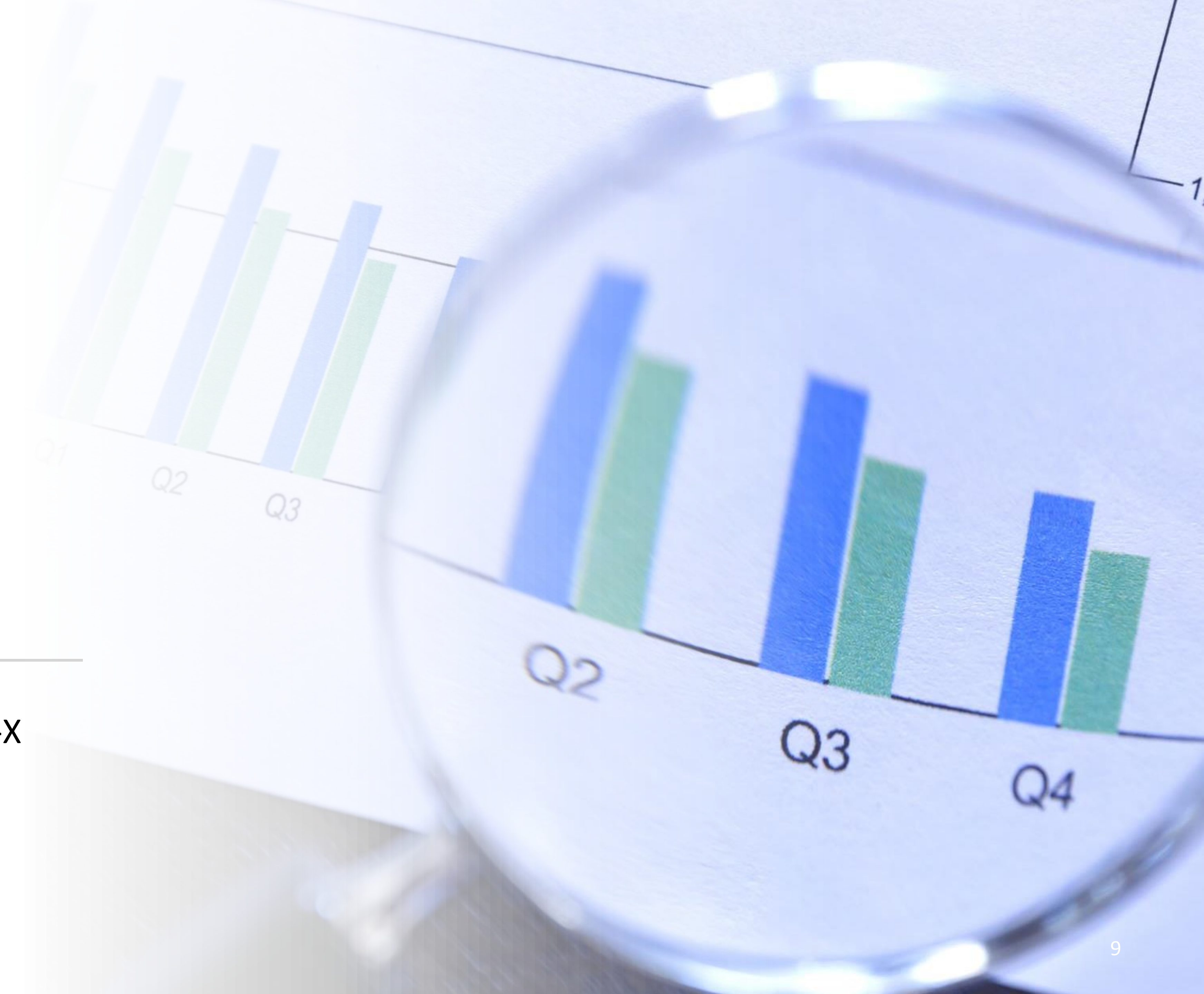

Background Information on CHEM-X

- Pesticide
- Occupational use only (small amounts detected in food)
- Primary exposure route = dermal; secondary = inhalation, ingestion
- Rapidly broken down and eliminated via urine. Little bioaccumulation (eliminated ~ 12 days)
- No evidence of carcinogenicity in animals (2-year oral cancer bioassay in rodents).

LEUKEMIA

Proposals

The Safety-First Fund received three proposals for observational epidemiological investigations to study the **potential association between exposure to the pesticide CHEM-X and leukemia**:

- Case Control
- Case Control Nested in Cohort (Semi-Ecologic)
- Cohort

Your goal as a grant reviewer at SFF, is to review these three proposals, and to determine which research group should obtain funding, and why.

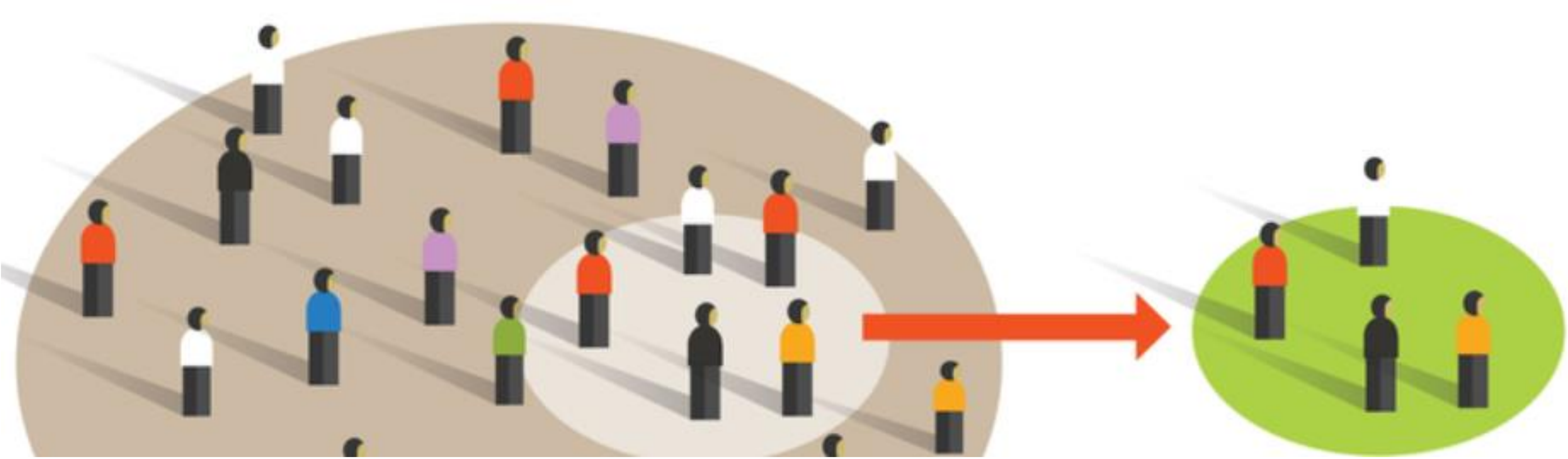

Note that for all proposals presented here, we will accept that:

- **All research teams have the same level of qualification**
- **Sample size was evaluated and determined to be adequate**

- *The risk tolerance level will be your own*

CASE CONTROL STUDY

Cases and Controls Selection

CASE CONTROL

CASES

Tumor Registry and Hospital Records

Pathology slides will be reviewed for all cases

Newly diagnosed cases of leukemia among white men aged 30 years or older

CONTROLS

Random digit dialing → Living controls <65 y.o.

Medicare Records → Living controls >65 y.o.

State Death Certificates → Deceased controls

White men without lymphatic cancer → Matching by 5-yr age groups, vital status at time of interview, state of residence

CASE CONTROL

Exposure Evaluation (1/3)

- **In-person** interviews (~50 min) will be conducted **with the subjects or with close relatives** if the subjects are deceased or unable to be interviewed.
- Standard questionnaire will be administered to collect detailed information about:
  - Residential history
  - Drinking water sources
  - Non-farm occupational history
  - Smoking and alcohol use
  - Use of unpasteurized dairy products
  - Medical conditions
  - Family history of cancer
  - Farm activities

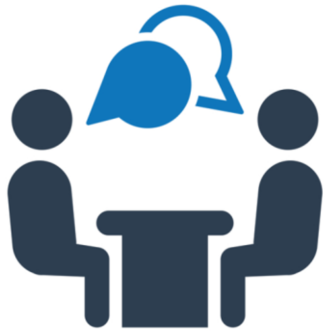

Exposure Evaluation (2/3)

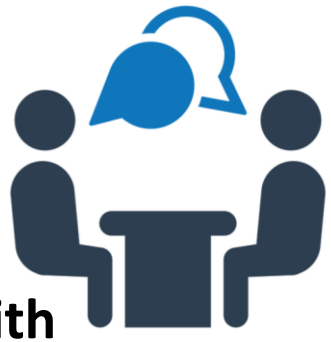

CASE CONTROL

- **In-person** interviews (~50 min) will be conducted **with the subjects or with close relatives** if the subjects are deceased or unable to be interviewed.
- Standard questionnaire will be administered to collect detailed information about:
  - Residential history
  - Drinking water sources
  - Non-farm occupational history
  - Smoking and alcohol use
  - Use of unpasteurized dairy products
  - Medical conditions
  - Family history of cancer
  - Farm activities

- Farm activities
  - Farm location
  - Number and type of animals and crops raised
  - Use of 24 animal insecticides, 38 herbicides and 16 fungicides used on the farm
    - First and last year used
    - Did the subject personally mixed or applied the pesticide?
    - Usual number of days per year that each pesticide was used

CASE CONTROL

Exposure Evaluation (3/3)

- Exposure will be quantified according to the following categories:
  - Ever farmed vs. never farmed
  - Type of crop grown
  - Acres of crop grown
  - Years farmed
    - 1-9 yrs.
    - 10-29 yrs.
    - 30-44 yrs.
    - 45+ yrs.
  - Used any fungicide (Ever-Never)
  - Used any insecticide (Ever-Never)
  - Used any herbicide (Ever-Never)
  - Day/yr. the pesticides were handled
    - 1-4 d.
    - 5-9 d.
    - 10+ d.

Statistical Analysis

CASE CONTROL

- **Odds-ratios** will be calculated to compare farmers with the exposure of interest with non-farmers, and **95% CI** will be calculated.
  - OR will be calculated for all leukemia and for each leukemia cell types, if the number of exposed subjects is sufficient
- **Confounding**
  - All models will be adjusted for vital status (alive, dead), age (<45, 45-64, >65 years), state, ever used tobacco daily (yes, no), parent, sibling, or child with a lymphopoietic cancer (yes, no), nonfarming job related to risk of leukemia in this study (yes, no), and exposure to substances (benzene, naphtha, hair dyes) related to risk of leukemia in this study (yes, no)
- **Bias**
  - Bias analysis will be conducted to account for latent (unmeasured) confounding

CASE CONTROL

Output

- **Publication**
  - Results will be published in a peer-reviewed epidemiology journal
  - Additional results that do not appear in the main article will be published as supplemental material

- **Data Sharing**
  - Collaboration with external investigators
    - All raw data will be stored in a publicly accessible repository while balancing protection of personally identifiable information
    - The detailed study protocol will be publicly accessible

- **Communication**
  - Results will be communicated to pesticide users and trade associations via townhall meetings and online webinars

CASE-CONTROL STUDY NESTED IN A COHORT STUDY* (SEMI-ECOLOGIC)

**Case control study within a retrospective cohort*

C-C NESTED IN COHORT STUDY

Cases and Controls Selection

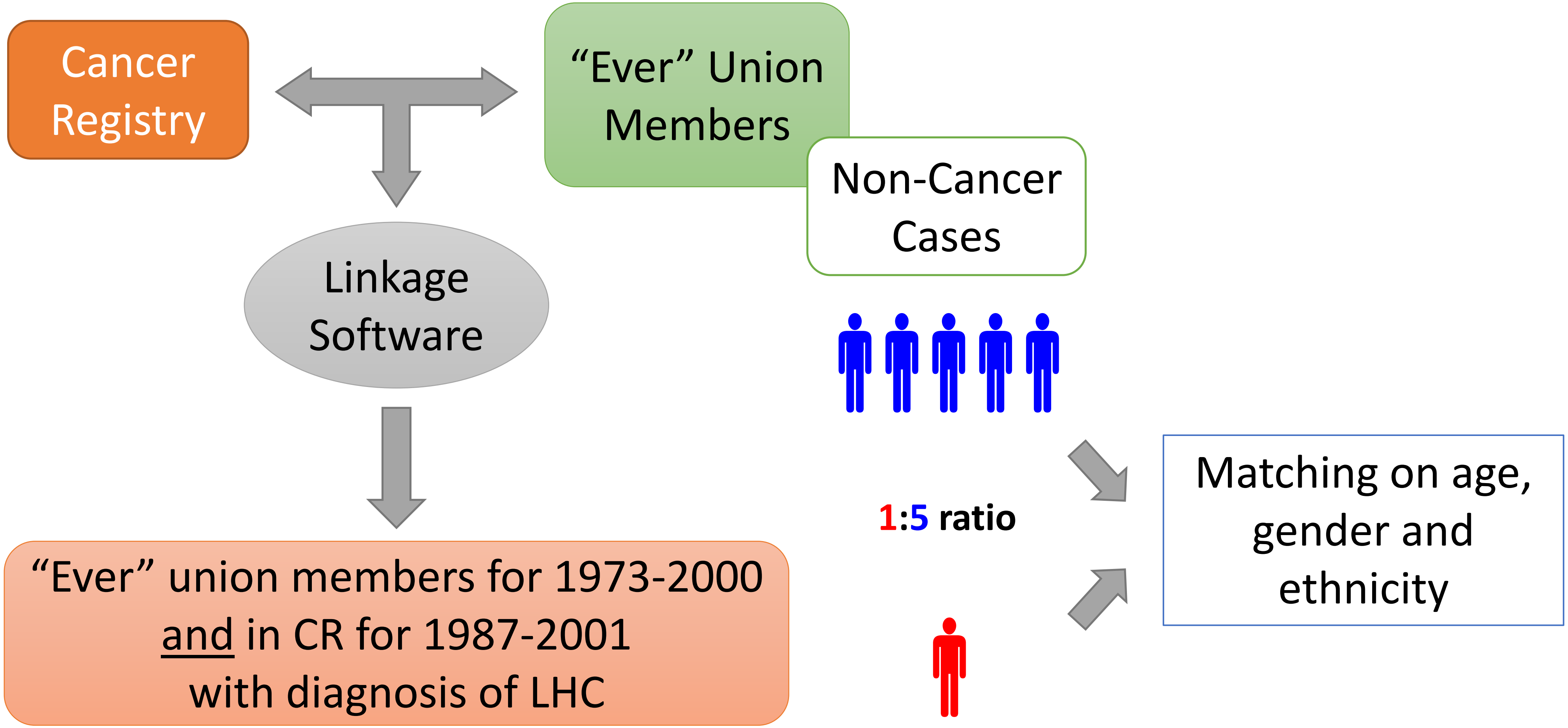

Exposure Evaluation (1/3)

- Will look at **exposure 20 years before cancer diagnosis**
- Will compile information from the **workers' union available on a month-to-month basis**
  - Duration of union membership
  - Duration of employment by different growers
  - Nature and location of the crops produced by each growers
  - Geographical location of employment

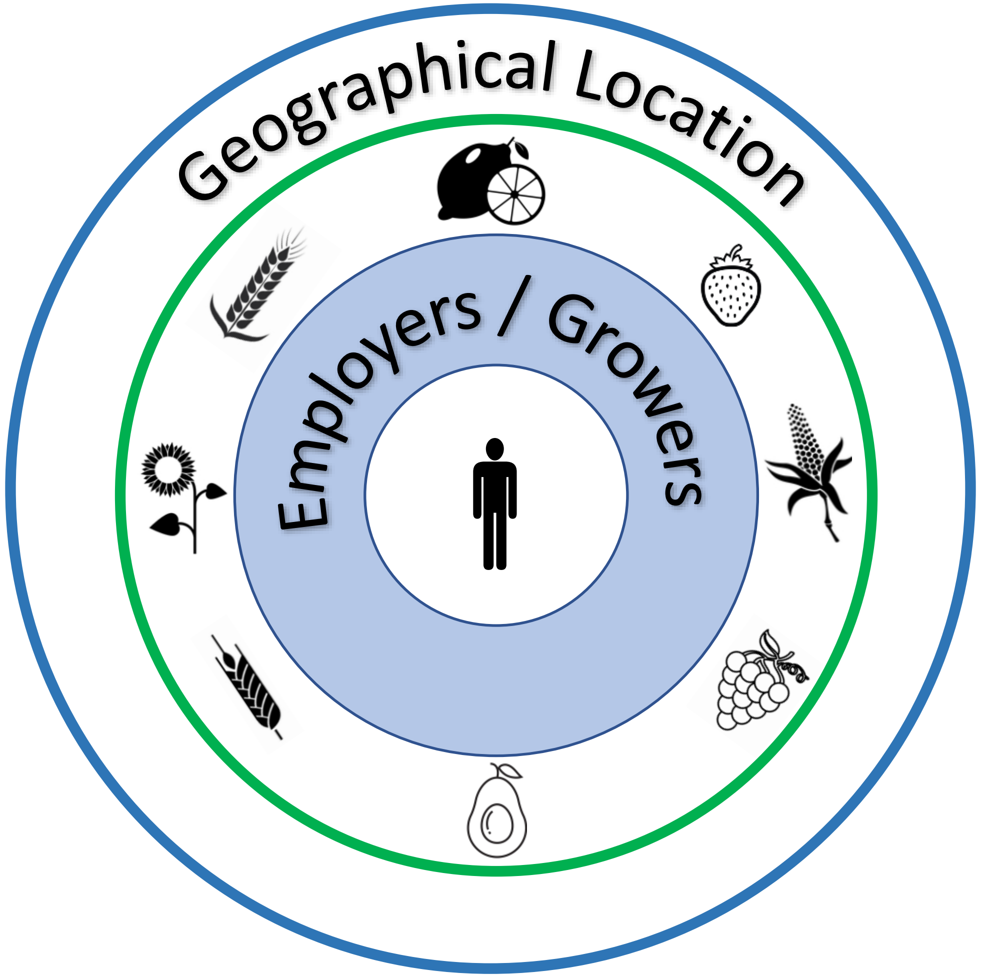

Exposure Evaluation (2/3)

- Looked at exposure 20 years before cancer diagnosis
- Information from the workers' union available on a month-to-month basis
- **Ecological data will be introduced in the study as information from the State Historical Pesticide Database**
  - County of use
  - Crop
  - Months/year of application
  - Area treated
  - Lbs. of A.I. applied

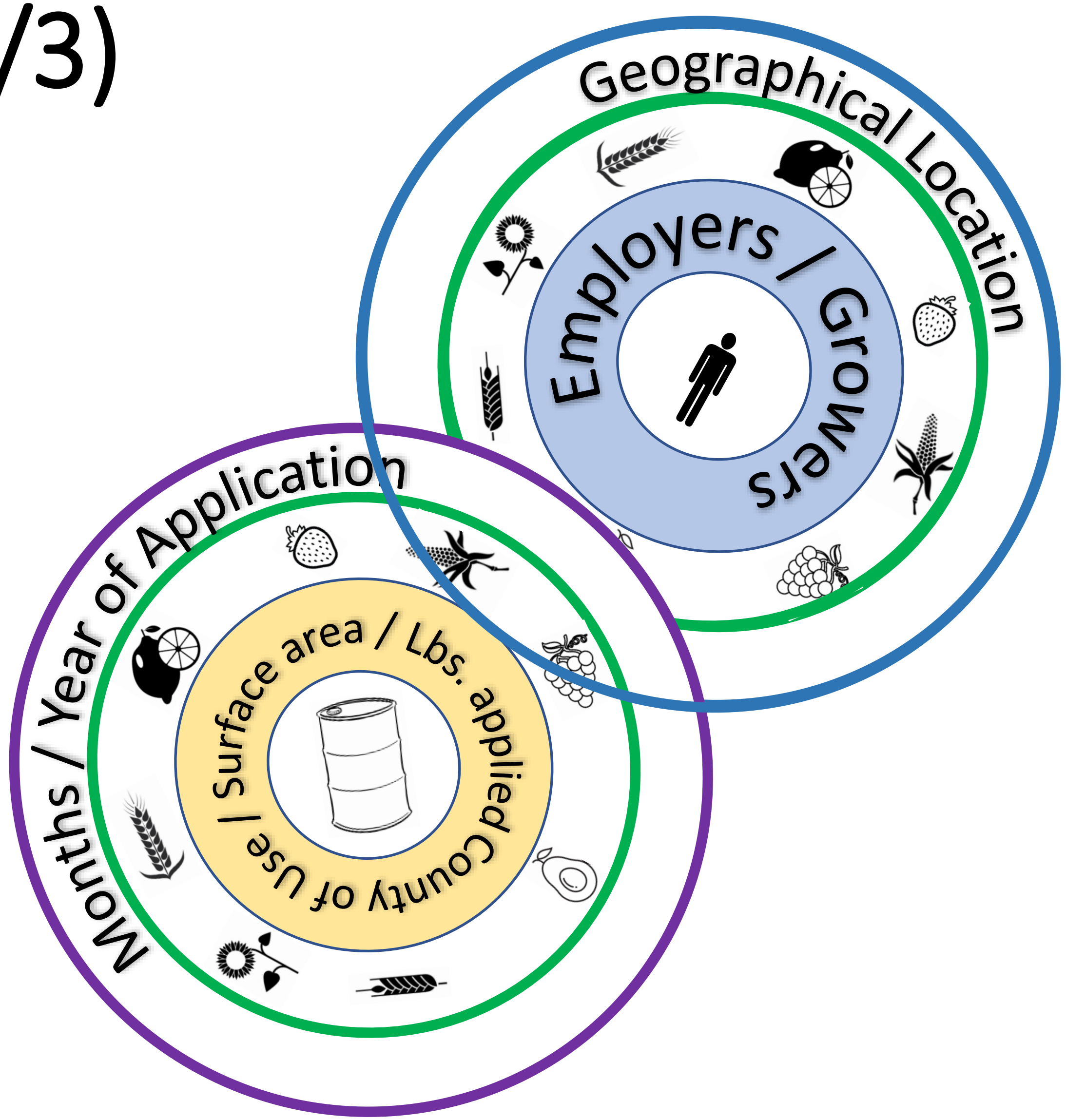

C-C NESTED IN COHORT STUDY

Exposure Evaluation (3/3)

- Looked at exposure 20 years before cancer diagnosis
- Information from the workers' union available on a month-to-month basis
- Information from the State Historical Pesticide Database (Ecological Information)

Exposure Proxy:

For any given crop, in a given month/year, and a given county, employment was matched to the corresponding pesticide application.

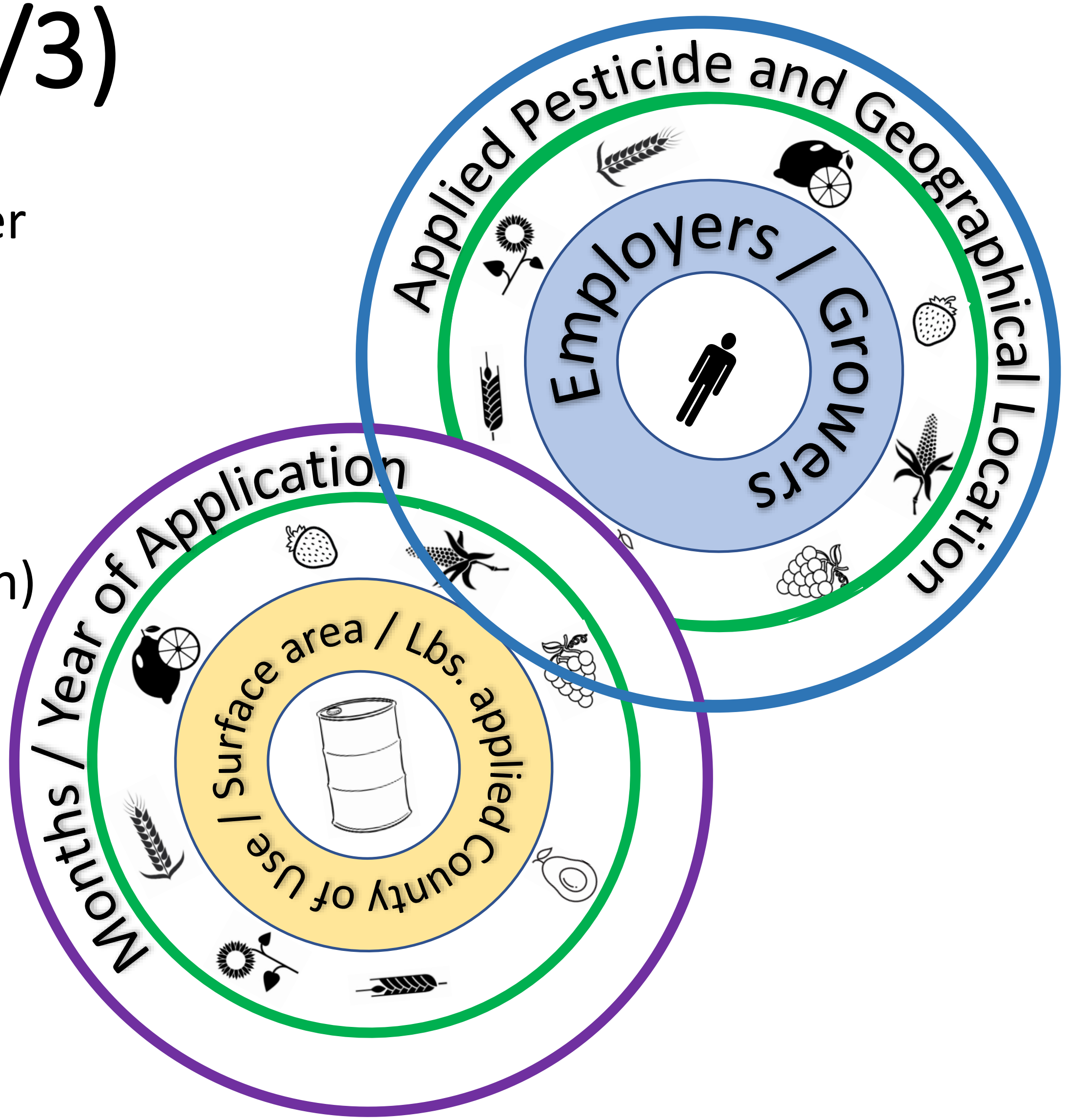

C-C NESTED IN COHORT STUDY

Statistical Analysis

- Lymphohematopoietic cancer cases and controls will be **stratified on age and sex**
- **Adjusted odds-ratios associated with different occupational variables** will be calculated using the method of Mantel and Haenszel
- **95% CI and trend p-values** will be calculated for ordinal variables
  - ORs will be calculated for all leukemia and for the leukemia cell types, if the number of exposed subjects is sufficient
- **Confounding**
  - Age, race/ethnicity and sex will be accounted for.
  - No information will be obtained on smoking history, diet or medical history, all of which may be involved in the etiology of LHC.

C-C NESTED IN COHORT STUDY

Output

- **Publication**
  - Results will be published in a peer-reviewed epidemiology journal
- **Data Sharing**
  - No data/protocol sharing
- **Communication**
  - Results will be communicated to pesticide users and trade associations via townhall meetings and online webinars

COHORT

Cohort Enrollment (1/2)

COHORT

Licensed pesticide applicators in 2 states

Sub-set following a mandatory certification sessions for pesticide use were given a take-home exposure evaluation questionnaire

Matching

Cancer Registry

All incident cancer cases diagnosed within study period will be included

Cohort Enrollment (2/2)

COHORT

- Vital status verification:
  - Through annual matching of cohort members to the National Death Index
- Residence status verification through:
  - Current address records of the Internal Revenue Service
  - Motor vehicle registration offices
- Follow-up will be censored at the time of participant death or movement out of the state.
- All participants will be asked to provided informed consent

Exposure Evaluation (1/3)

COHORT

- Exposure will be evaluated through **enrollment and take-home, self-administered questionnaires and calibrated via measurement of CHEM-X metabolites in 200 urine samples prospectively collected**

ENROLLMENT QUESTIONNAIRE

Will collect information on:

- **Ever/never use of 50 commonly used pesticides**
- **Detailed information on lifetime exposure for 22 pesticides**
  - Number of days per year and number of years
  - Pesticide application and mixing methods
  - Repair of equipment
  - Use of personal protective equipment.

- **Potential risk factors** such as:
  - Smoking,
  - Alcohol consumption
  - Cancer history of first-degree relatives
  - Diet
  - Selected medical conditions
  - Demographic information

Exposure Evaluation (2/3)

- Exposure will be evaluated through **enrollment and take-home, self-administered questionnaires and calibrated via measurement of CHEM-X metabolites in 200 urine samples prospectively collected**

SELF-ADMINISTERED TAKE-HOME QUESTIONNAIRE

Will collect information on:

COHORT

- Participants will provide information on **duration of exposure** for the remaining 28 pesticides, **including CHEM-X**

Exposure Evaluation (3/3)

COHORT

- CHEM-X exposure will be quantified according to the following categories:

- **Non-exposed / Lowest exposed/Highest exposed**

- **Duration of exposure** (days):
  - 0 d.
  - <20 d.
  - 20-38.8 d.
  - >38.8 d.

- **Cumulative exposure** (days)*:
  - No exposure
  - Tertile 1
  - Tertile 2
  - Tertile 3

(* Accounts for pesticide handling methods)

COHORT

Statistical Analysis (1/3)

- Two referent groups will be used for all studies:
  - **Report of no use of CHEM-X**
  - **Lowest tertile of CHEM-X use**

⇨ *This will be done because uncontrolled confounding could occur if the nonexposed group is different from the high exposed group regarding unmeasured (and therefore uncontrollable) risk factors.*

- Analyses will be stratified by sex and minimum samples size (case counts), and will exclude those who had a cancer diagnosis prior to enrolling in the study.

COHORT

Statistical Analysis (2/3)

- **A Poisson regression will be used to calculate rate ratios**
- A **test for linear trend** will be performed using the median of each exposure category as the quantitative score.
- All **tests for significance** will be two-sided
- **Results will be adjusted for errors in exposure evaluation** using information from calibration studies based on biomonitoring (urine) data

Statistical Analysis (3/3)

COHORT

- **Confounding:** All rate ratios will be adjusted for
  - Age as a categorical variable
  - Smoking history
  - Alcohol consumption
  - Education
  - State of residence
  - Family history of cancer, and
  - Lifetime days of any pesticide application (continuous variable based on days and years of use).

- **Bias**
  - Results will be corrected for potential bias from self-reports
  - The impact of selection bias (e.g., differential loss to follow-up) on the effect size estimate will be estimated

COHORT

Output

- **Publication**
  - Results will be published in a peer-reviewed epidemiology journal
- **Data Sharing**
  - All raw data will be stored in a publicly accessible repository
  - The detailed study protocol will be publicly accessible
- **Communication**
  - No plan

CASE CONTROL STUDY

SEMI-ECOLOGICAL STUDY

COHORT

Summary

LEUKEMIA	Case-Control	C.C Nested in Cohort	Cohort
**Sample size**	Sample size was evaluated and determined to be adequate for all studies		
**Design**	✓ Tumor registries/Hospital records + confirmation with pathology slides	✓ Tumor registry linked with occupational cohort (Union membership)	✓ Tumor registry linked with occupational cohort study (licensed pesticide applicators)
**Exposure**	✓ Self-Report via Questionnaire<br>▪ Pesticide-Specific Questions<br>▪ Ever-Never Pesticide Use<br>▪ Lifetime Pesticide Use<br>● No Exposure Validation	✓ Indirect Exposure Surrogate based on County Pesticide Use Records<br>● Ecological Measure of Exposure<br>● No Exposure Validation	✓ Self-Report via Questionnaire<br>▪ Pesticide-Specific Questions<br>▪ Ever-Never Pesticide Use<br>▪ Lifetime Pesticide Use<br>✓ Validity of exposure assessment evaluated
**Analysis**	✓ Detailed consideration of covariates<br>● No analysis of bias	● Limited consideration of covariates<br>● No analysis of bias	✓ Detailed information on covariates<br>✓ Bias and sensitivity analysis performed<br>✓ Reliability study performed
**Output**	✓ Peer-Reviewed Journal Publications<br>✓ Protocol/Supplemental Info Available<br>✓ Data Publicly Available<br>✓ Townhall Meetings	✓ Peer-Reviewed Journal Publications<br>✓ Townhall Meetings<br>● No Protocol/Supplemental Info<br>● Data not accessible	✓ Peer-Reviewed Journal Publications<br>✓ Protocol/Supplemental Info Available<br>✓ Townhall Meetings<br>● Data not accessible
**Budget**	**$$ ($450,000)**	**$ ($250,000)**	**$$$$ (>$1M/yr.)**

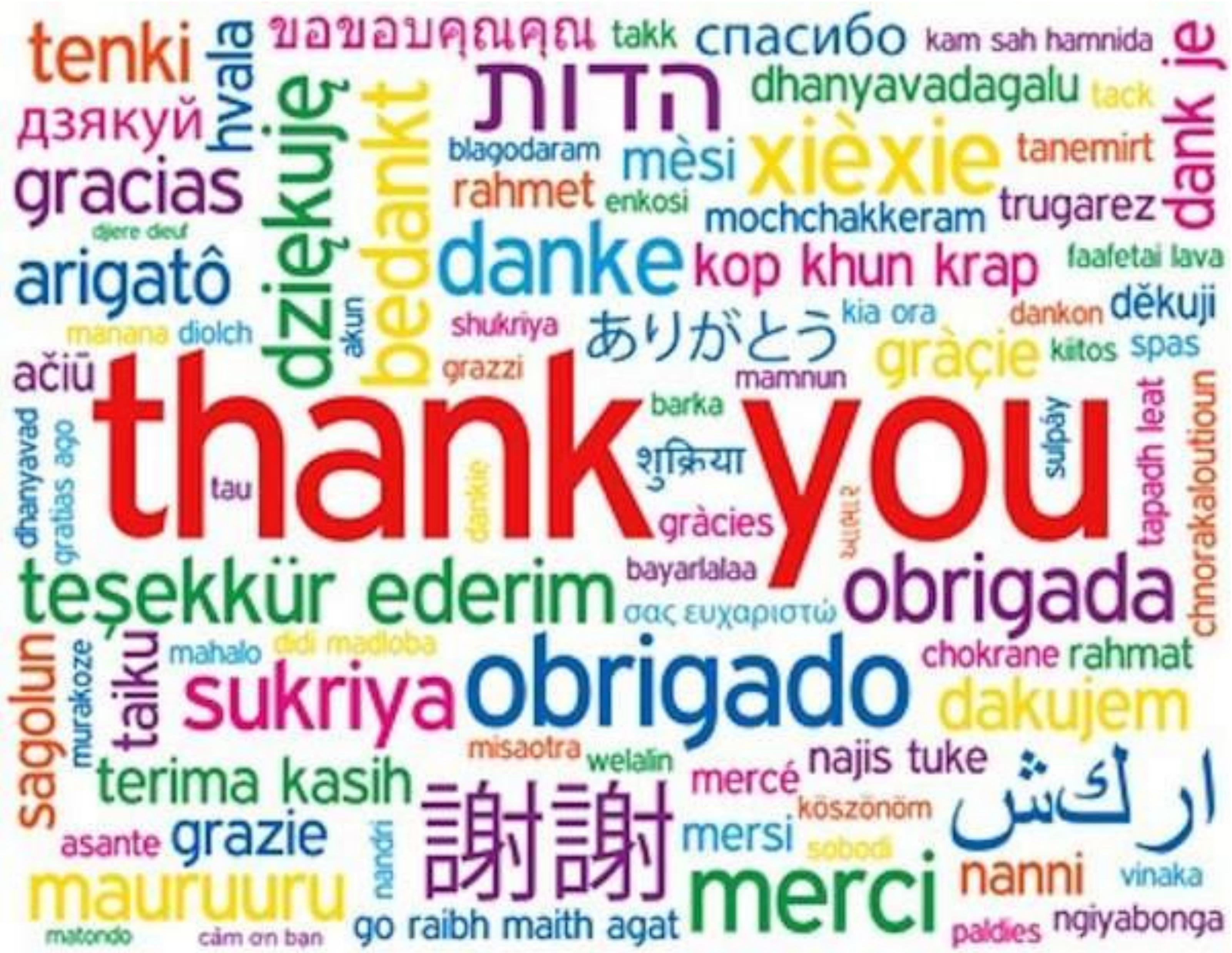

[This Photo](#) by Unknown Author is licensed under [CC BY-NC-ND](#)

DRAFT - Do not circulate or cite.

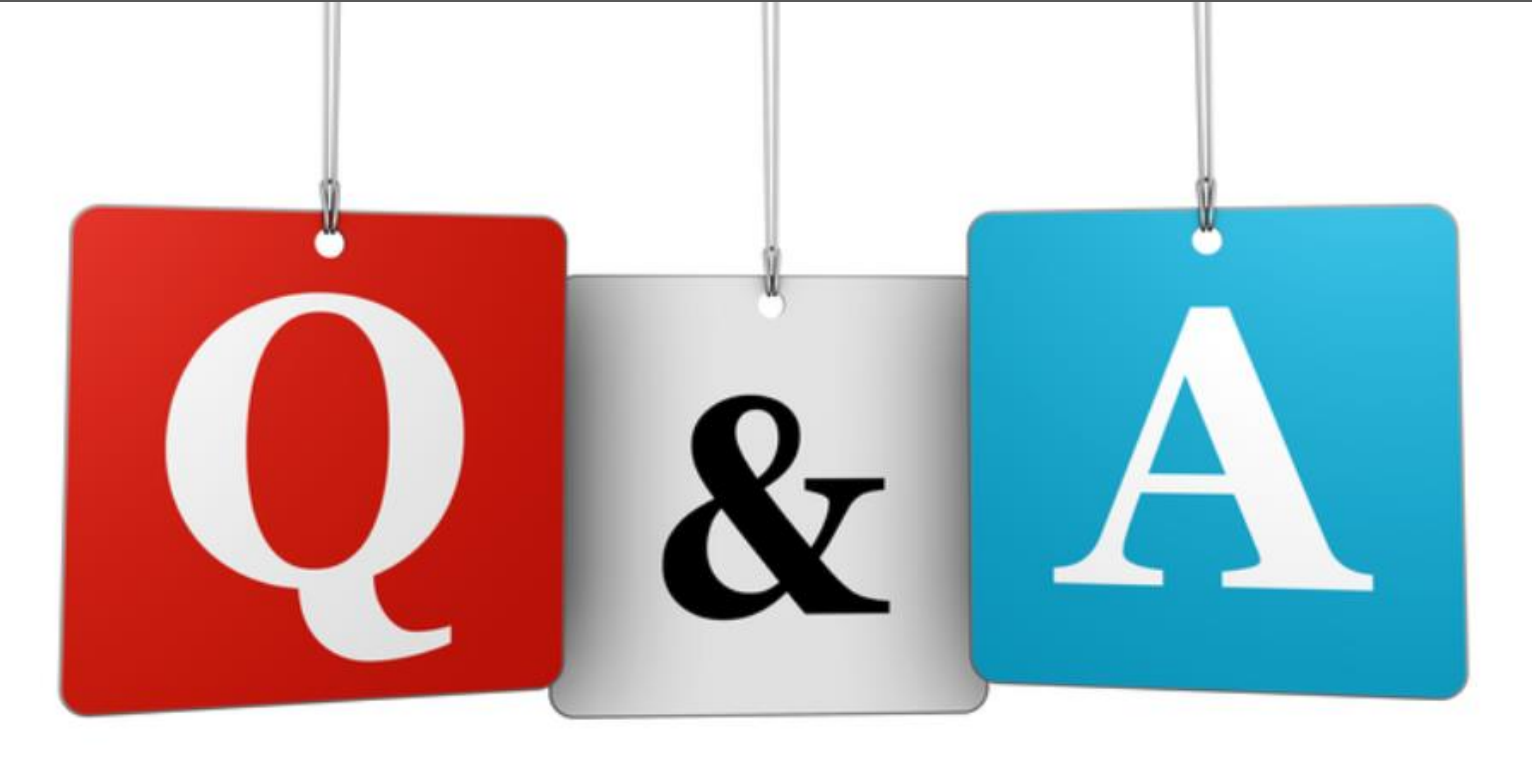
Q
&
A
